# Mechanical power output during stretch–shortening cycles of rat medial gastrocnemius muscle: Influence of various muscle length trajectories

**DOI:** 10.1371/journal.pcbi.1014587

**Published:** 2026-08-12

**Authors:** Edwin D. H. M. Reuvers, Huub Maas, Wendy Noort, Maarten F. Bobbert, Dinant A. Kistemaker

**Affiliations:** Department of Human Movement Sciences, Faculty of Behavioural and Movement Sciences, Vrije Universiteit Amsterdam, Amsterdam Movement Sciences, The Netherlands; Imperial College London Faculty of Engineering, UNITED KINGDOM OF GREAT BRITAIN AND NORTHERN IRELAND

## Abstract

The average mechanical power output (AMPO) over a stretch–shortening cycle produced by a muscle depends on muscle length and stimulation over time. While the effects of cycle frequency and muscle length excursion on AMPO are well-known, several questions remain about the effects of muscle length and stimulation over time on the maximally attainable AMPO. For example, which precise muscle length and stimulation over time yield maximal AMPO? *In situ* experiments are inherently limited to a finite set of muscle length and stimulation over time. To overcome this limitation, we combined *in situ* experiments on rat *m. gastrocnemius medialis* with Hill-type muscle modelling. We first performed dedicated trials to estimate the muscle-tendon-complex (MTC) properties of each rat. Subsequently, we performed various stretch–shortening cycles with substantial differences in cycle frequency, shortening-to-lengthening time ratio and MTC length excursion. Model-predicted AMPO correlated nearly perfectly with experimentally measured AMPO (r² > 0.98). This justified further exploration using the Hill-type MTC model. Using the Hill-type MTC model, we predicted that AMPO peaks at a cycle frequency of 3.5 Hz, with a shortening-to-lengthening time ratio of 6:1 and an MTC length excursion of 8 mm. Notably, cycle frequency and MTC length excursion showed a strong interaction: increasing one necessitated a decrease in the other to maximise AMPO. By contrast, the optimal shortening-to-lengthening time ratio remained remarkably constant across all tested combinations of cycle frequency and MTC length excursion. This shows that muscles should spend substantially more time shortening than lengthening to maximise AMPO.

## Author summary

The average mechanical power output (AMPO) of cyclic contractions, in which a muscle shortens and lengthens, is a key determinant of performance in tasks like sprint cycling and rowing. Apart from stimulation, muscle AMPO depends on three key factors: cycle frequency (the number of shortening–lengthening cycles per second), length excursion (the change in muscle length during each cycle), and the relative duration of shortening and lengthening. To study the dependency of AMPO on these key factors, we combined experiments on rat calf muscle with a mathematical muscle model incorporating key physiological properties. The model accurately reproduced the experimental results, which allowed us to explore a much wider range of the three factors than would be possible using experiments alone. Cycle frequency and length excursion interacted strongly, such that increasing one required decreasing the other to maximise AMPO. By contrast, the optimal relative duration of shortening and lengthening was consistent across all tested cycle frequencies and length excursions: AMPO was maximised when muscles spent about 85% of each cycle shortening. These findings improve our understanding of muscle function and may help explain animal behaviour as well as inform sports equipment design to enhance performance.

## Introduction

In motor activities such as cycling, rowing, and speed skating, muscles periodically shorten and lengthen, a pattern commonly referred to as the stretch–shortening cycle (SSC). When muscles produce force while shortening, they deliver positive mechanical power, and when they produce force while lengthening, they deliver negative mechanical power. During motor activities, part of the mechanical energy delivered by the muscles is dissipated to the environment, and this energy loss generally increases with movement speed. Consequently, performance critically depends on the average mechanical power output (AMPO) that muscles can produce over these SSCs. This raises several fundamental questions: how does muscle length over time influence the maximally attainable AMPO? More specifically, what are the effects of cycle frequency and muscle length excursion? Which fraction of the cycle should be spent shortening? And (how) do these factors interact? Surprisingly, despite the importance of AMPO for movement performance, many of these questions have not yet been answered.

**Fig 1:**
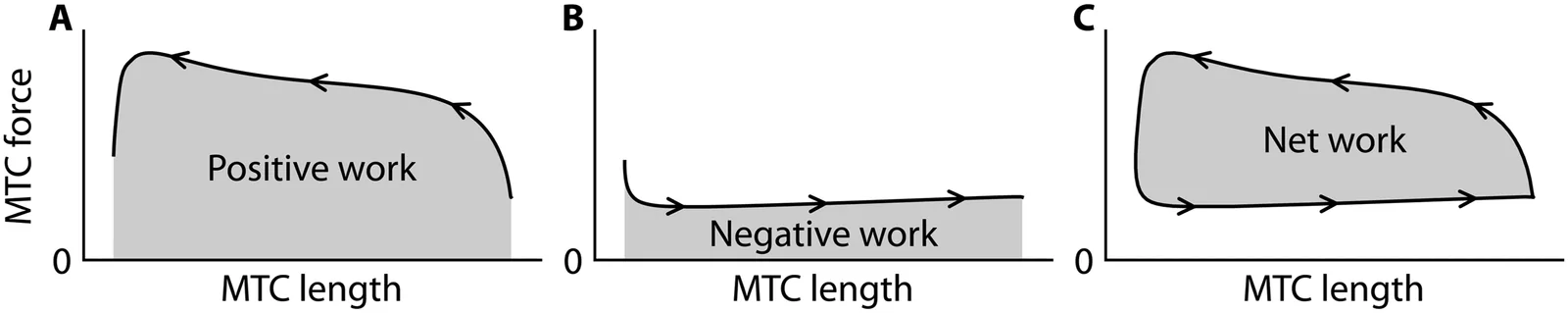
An example of a work loop. In a work loop, MTC force is plotted against MTC length (change). The area enclosed by the work loop represents the net mechanical work produced during a full cycle **(C)**, which is the sum of the positive mechanical work during MTC shortening (A) and the negative mechanical work during MTC lengthening **(B)**. The arrows indicate the direction of the work loop over time.

To study the mechanical output over an SSC, researchers use an experimental setup in which a motor imposes cyclical length changes on either isolated muscle fibres or the entire muscle–tendon complex (MTC). While the approach is the same at both levels, this paper focuses on SSCs at the MTC level. During each SSC, the MTC first undergoes lengthening and then shortens back to its initial length. Muscle stimulation is applied during part of the SSC, while MTC force is measured. When MTC force is plotted against MTC length (change), the area enclosed by the resulting loop represents the net mechanical work produced during a full cycle. This net mechanical work is the sum of the positive mechanical work during MTC shortening (Fig 1A) and the negative mechanical work during MTC lengthening (Fig 1B). This method has been coined the ‘work loop technique’ [1]. The average mechanical power output (AMPO) for each cycle can then be calculated by dividing the net mechanical work per cycle by the duration of the cycle. As such, the work loop technique provides a powerful tool to study the effect of MTC length and stimulation over time on the maximally attainable AMPO of muscles.

The work loop technique has been extensively employed to examine how MTC (or muscle fibre) length and muscle stimulation over time affect the mechanical behaviour of muscles. In most experiments, SSCs have been studied using sinusoidal length changes [e.g., 1]. These SSCs are typically parametrised in terms of cycle frequency and MTC/muscle fibre length excursion, with length changes centred around the length yielding maximal isometric force. Furthermore, muscle stimulation is typically parametrised in terms of stimulation onset time and stimulation duration (i.e., ‘duty cycle’). Since muscle stimulation substantially affects the mechanical behaviour of muscles [e.g., 2–4], stimulation onset time and duration are typically optimised to maximise mechanical work per cycle. This approach allows for systematically investigating the effects of cycle frequency, MTC/muscle fibre length excursion, and their interaction on AMPO. For example, results from such experiments have demonstrated a substantial interaction between cycle frequency and MTC/muscle fibre length excursion, whereby maximising AMPO typically requires larger length excursions at lower frequencies and smaller length excursions at higher frequencies [e.g., 5–7]. Although sinusoidal SSCs have experimentally provided valuable insights, they inherently impose equal shortening and lengthening durations, leaving unexplored how unequal shortening and lengthening durations affect AMPO.

**Fig 2:**
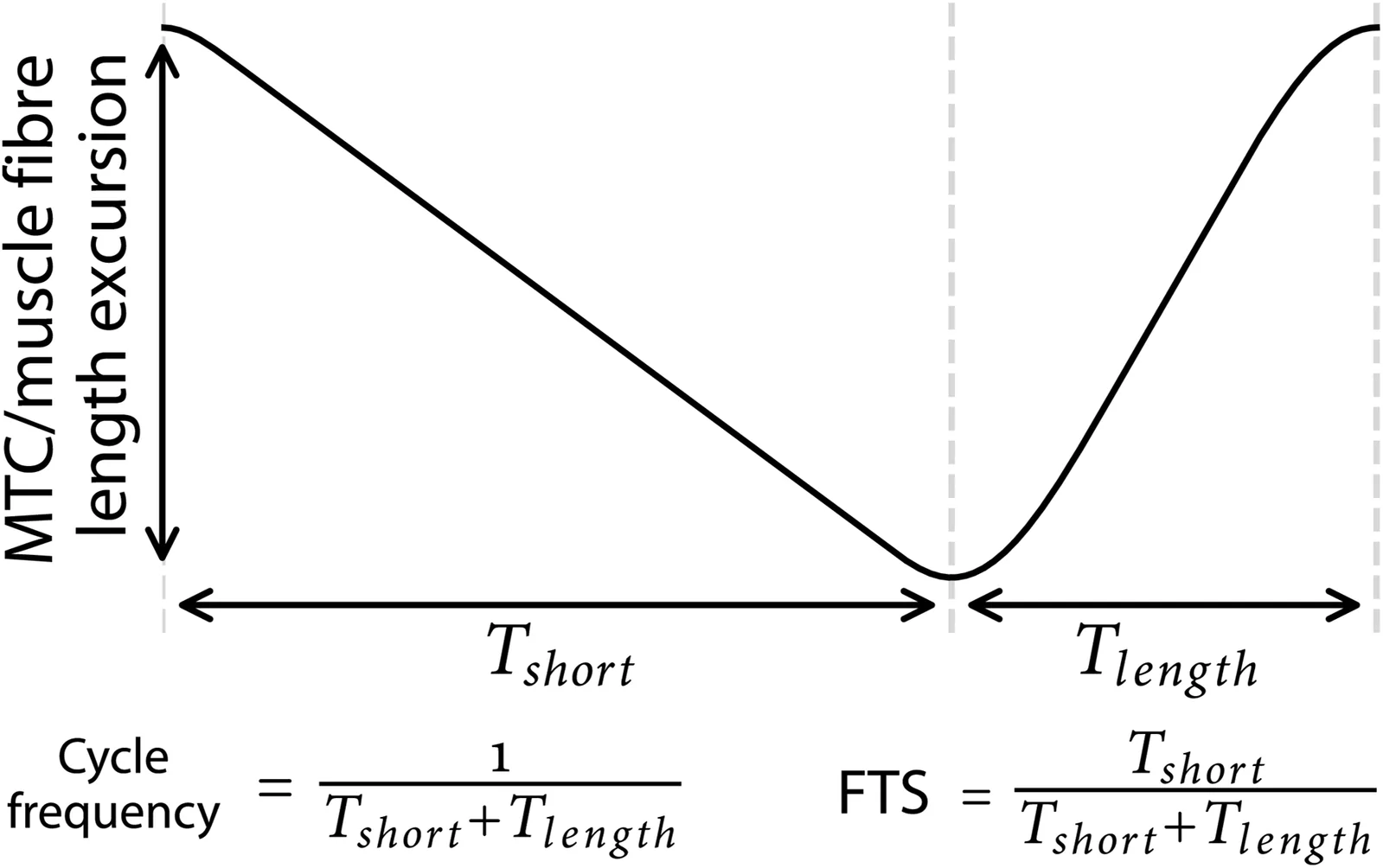
Representation of the parameterisation of stretch–shortening cycles. $T_{short}$ and $T_{length}$ denote the shortening and lengthening durations, respectively, of either the muscle-tendon-complex (MTC) or the muscle fibres. FTS denotes the fraction of the cycle time spent shortening. In the example shown, the MTC/muscle fibres shorten 65% of the cycle duration (i.e., FTS = 0.65).

Askew & Marsh [8,9] performed ground-breaking experiments on the effect of unequal shortening and lengthening durations on the maximally attainable AMPO — remaining, to our knowledge, the only researchers to have done so. In their studies, mouse *m. soleus* and *m. extensor digitorum longus* were connected at the distal part of the muscle belly to a servomotor for precise control of the imposed SSCs. The SSCs used by Askew & Marsh were parameterised using three variables (see Fig 2): cycle frequency, FTS (fraction of the cycle time spent shortening), and muscle fibre length excursion (i.e., maximum minus minimum fibre length), with near-constant shortening and lengthening velocities. Askew & Marsh [8,9] examined three distinct FTS values — 0.25, 0.50, and 0.75 — and heuristically optimised muscle fibre length excursion, muscle stimulation onset time and muscle stimulation duration to achieve the highest AMPO at different combinations of cycle frequency and FTS. Their findings indicated that AMPO was highest for FTS = 0.75, followed by FTS = 0.50, and lowest for FTS = 0.25 at each cycle frequency at the optimal muscle fibre length excursion and muscle stimulation.

While Askew & Marsh’s experimental studies provided valuable insights, several questions remain regarding the effect of SSC parameters on the maximally attainable AMPO. First, Askew & Marsh [8,9] optimised muscle fibre length excursion for each cycle frequency at only three distinct FTS values. Therefore, it remains unclear how muscle fibre length excursion affects the maximally attainable AMPO at different FTS values. More generally, it remains unclear how sensitive the maximally attainable AMPO is to variations in SSC parameters. For instance, does AMPO substantially decrease with suboptimal SSC parameters, or is there a wide range of SSC parameter combinations that yield near-maximal AMPO? Second, because Askew & Marsh [8,9] only used three distinct FTS values, it remains unclear how cycle frequency and muscle fibre length excursion affect the optimum value of FTS. More generally, it remains unclear how the optimum SSC parameters depend on each other. Third, Askew & Marsh [8,9] imposed near-constant muscle fibre shortening and lengthening velocities. Consequently, it remains unclear how much more AMPO the muscle could produce if the muscle fibre length over time would be optimal for AMPO. For this, the optimal muscle fibre length and stimulation over time should be identified in a parameter-free manner.

While it is obvious that muscle fibres should maximise positive mechanical work and minimise negative mechanical work in order to maximise AMPO, it is far from trivial what SSC and muscle stimulation over time yield maximal AMPO. To minimise negative mechanical work, stimulation should cease well before lengthening begins (because of deactivation time delays) and muscle fibres should ideally lengthen at very low velocities (as muscle fibre force increases with increasing lengthening velocity). However, such strategies are suboptimal for AMPO: the time spent lengthening cannot be used to produce positive mechanical work, and early deactivation reduces the positive mechanical work during shortening. One might argue that muscle fibres should shorten at a velocity that maximises the instantaneous mechanical power output to maximise positive mechanical work. However, muscle fibre force also depends on muscle fibre length, with muscle fibre force decreasing at both shorter and longer lengths than optimum length. Therefore, to maximise AMPO, it may be beneficial for muscle fibres to shorten at a velocity lower than that maximising instantaneous mechanical power output. Doing so limits the negative effects of operating at suboptimal muscle fibre lengths. This raises several open questions: What shortening velocity maximises AMPO? Over what length range should muscle fibres operate? And how much time should be spent on shortening (to maximise positive mechanical work) versus lengthening (to minimise negative mechanical work) in order to maximise AMPO?

Obviously, it is hard to experimentally find the optimal combination of cycle frequency, FTS, MTC length excursion and muscle stimulation for maximal AMPO. If one would change MTC length excursion, how should the other SSC parameters and muscle stimulation be changed to maximise AMPO? Or, taking it one step further: How should we find the MTC length and stimulation over time that maximises AMPO when the muscle length over time is entirely free to take any form? For this reason, we propose to complement *in situ* experiments with simulations and optimisations using a Hill-type MTC model to study the effects of MTC length and muscle stimulation over time on the maximally attainable AMPO. If the MTC model yields accurate predictions of the influence of SSC parameters and muscle stimulation over time on the maximally attainable AMPO, it would allow us to answer a plethora of questions regarding the maximally attainable AMPO of various SSCs. For instance, we could investigate how the optimum SSC parameters depend on each other, and how sensitive the maximally attainable AMPO is to variations in SSC parameters. Furthermore, using an accurate model, we could optimise MTC length over time for maximal AMPO and quantify how much more AMPO could be produced compared to sinusoidal SSCs or the near-constant velocity SSCs used by Askew & Marsh [8,9].

In this study, we aimed to explore the influence of SSC parameters — cycle frequency, FTS and MTC length excursion — on the maximally attainable AMPO of a single isolated MTC. Thus, we considered the maximally attainable AMPO for each combination of SSC parameters. To determine this, muscle stimulation was optimised for every SSC. As discussed earlier, only a limited set of SSCs can be investigated experimentally, so this raises the question: Which SSCs should be selected? To make an informed selection of SSCs to be tested experimentally, we first used a Hill-type MTC model — with parameter values obtained from literature — to simulate a broad range of SSCs. Based on these simulation results, we selected a smaller, representative and informative set of SSCs that captured the most prominent effects, providing a focused basis for evaluating model predictions. This set of SSCs was then used in an *in situ* experiment on rat *m. gastrocnemius medialis*. In this experiment, we also performed dedicated trials to estimate the MTC properties of each specimen individually. This allowed us to compare the experimental results with model-based predictions, which showed a nearly perfect correlation between measured and model-predicted AMPO (r^2^ > 0.98). This strong correspondence then justified the use of the Hill-type MTC model to (finally) explore how SSC parameters — cycle frequency, FTS and MTC length excursion — affect the maximally attainable AMPO and to identify the fully unconstrained optimal MTC length over time for maximal AMPO.

## Methods

### Ethics statement

All surgical and experimental procedures conducted in this study were approved by the Committee on the Ethics of Animal Experimentation at the Vrije Universiteit (Permit Number: FBW-AVD11200202114471) and adhered to Dutch law concerning the guidelines and regulations of animal welfare and experimentation.

### Study outline

We combined *in situ* experiments with simulations and optimisations using a Hill-type MTC model to investigate the influence of SSC parameters — cycle frequency, FTS, and MTC length excursion — on the maximally attainable AMPO of rat *m. gastrocnemius medialis* (GM). We first used a Hill-type MTC model with parameter values from literature to simulate a broad range of SSCs. Based on these simulation results, we selected a representative and informative subset for an *in situ* experiment, in which MTC length and stimulation over time were under full control. In this experiment, we also performed dedicated trials to estimate the MTC properties of each specimen individually. Model-based predictions were then evaluated against the experimental data. The strong correspondence (r^2^ > 0.98) justified the use of the Hill-type MTC model to explore the influence of SSC parameters on the maximally attainable AMPO across a broad SSC parameter space. First, we derived predictions for SSCs with constant MTC shortening and lengthening velocity. Second, we identified the optimal SSC without any constraints on MTC length over time to investigate how much more AMPO the muscle could produce if the muscle were allowed to shorten-and-lengthen optimally.

### *In situ* experimentIn situ experiment

#### Animals.

Data were obtained from three male Wistar rats (body mass 365, 410 and 407 gram, respectively). The rats were anaesthetised by intraperitoneally injecting a urethane solution (12.5% urethane) at a dosage of 1.2 mL/100 g body mass. Additional doses of urethane (0.2 mL/g body mass) were administered throughout the experiment as needed to maintain deep anaesthesia. Heart rate, body temperature and withdrawal reflexes to a pain stimulus were monitored every 10 minutes. To prevent dehydration, subcutaneous injections of 1 mL NaCl solution (0.9%) were administered every 1–2 hours. After completion of the experiment, we euthanised the rats by intracardially injecting pentobarbital sodium (Euthasol 20%) followed by a double-sided pneumothorax.

#### Surgical procedure.

The hindlimb of the rat was shaved, and the skin was cut and partially removed. Next, *m. biceps femoris* was removed to expose the femur. To minimise mechanical effects of surrounding muscles on GM — in particular myofascial force transmission — the following steps were taken [see 10]: 1) GM was exposed as much as possible from its surrounding tissue. 2) The tendons of *m. plantaris*, *m. gastrocnemius lateralis* and *m. soleus* were cut at their distal ends. 3) The medial and lateral parts of *m. gastrocnemius* were carefully separated. *N. peroneus*, *n. suralis*, and the branch of the *n. tibialis* innervating *m. gastrocnemius lateralis* and *m. soleus* were then cut to further reduce the influence of surrounding tissue on GM. Subsequently, the tissue around the calcaneal tendon was removed such that only a small part of the calcaneus was left. Lastly, a cuff-electrode was placed on *n. ischiadicus*. The part of *n. ischiadicus* proximal to the cuff-electrode was then crushed to prevent muscle excitation via spinal reflexes.

#### Experimental setup.

The rats were placed on an electrical heating pad to maintain body temperature at about 37 °C. The laboratory temperature was kept constant at 20 °C, with a local humidity around the muscle tissue of about 80%. A servomotor (Aurora 309C, Aurora Scientific, Aurora, Canada) was used to impose MTC length changes and to measure GM force. Before the experiment, we calibrated the position sensor of the servomotor using a dial gauge (2046S, Mitutoyo Nederland BV, Veenendaal, the Netherlands) and calibrated the force sensor using various calibration masses. To relate servomotor position (changes) to MTC length, we measured MTC length at one position with a caliper at the end of the experiment. A direct current stimulator was used to stimulate *n. ischiadicus* with a pulse width of 100 μs and at a frequency of 100 Hz during all experiments, while the (supramaximal) current was chosen for every rat individually. Before the series of experiments, we ensured that the position, force, and stimulation signals were synchronised in time by applying a physical stimulus that produced a detectable response across all channels. All data were collected at a sample rate of 2000 Hz.

**Fig 3:**
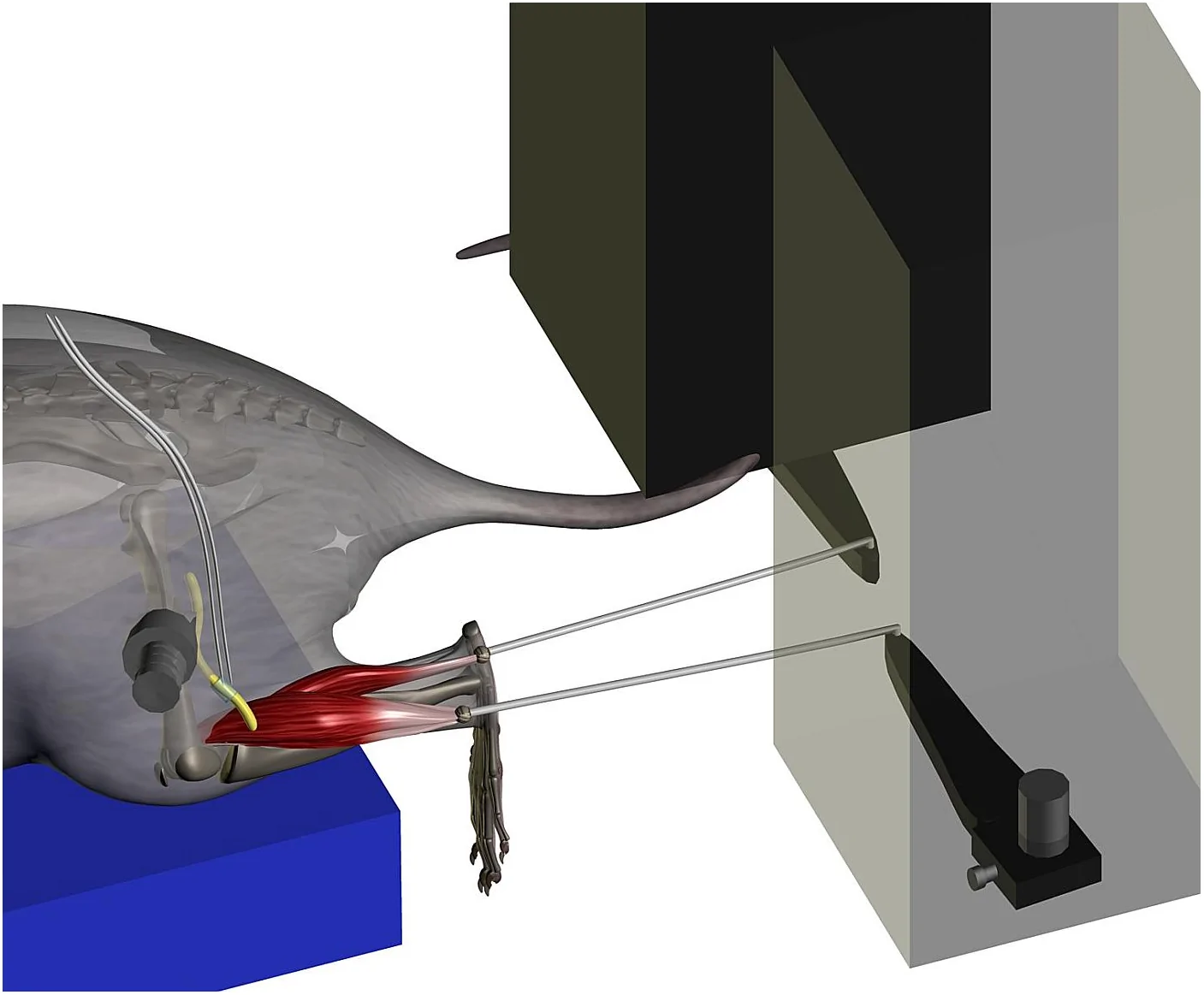
Representation of the experimental setup that provided full control of MTC length and stimulation while measuring *m. gastrocnemius medialis* (GM) force. GM was carefully exposed from its surrounding tissue and positioned in the setup such that GM pulled in its natural direction, while the femur and foot were securely fixated. The distal end of the calcaneal tendon was connected to a motor via a steel rod. The distal tendon of *m. gastrocnemius lateralis* and *m. plantaris* was connected to a second motor. A cuff-electrode was placed on *n. ischiadicus*. *N. peroneus*, *n. suralis* and the branch of *n. ischiadicus* innervating *m. gastrocnemius lateralis* and *m. soleus* were cut such that only GM was innervated.

The femur was fixed with a metal clamp and the foot with a plastic plate, such that the hindlimb was rigidly secured to the setup. The distal end of the calcaneal bone was tightly attached to a steel rod with 100% polyester yarn at the leftover part of the calcaneus. The other end of the steel rod was then attached with Kevlar thread to the motor. A similar procedure was applied to *m. gastrocnemius lateralis* (GL) and *m. plantaris* (PL). The distal tendon of GL and PL (GL + PL) was then connected to a second motor to monitor their combined force throughout the experiment in order to verify the absence of mechanical interaction with GM. Care was taken to ensure that the direction of GM force reflected *in vivo* conditions. Lastly, we connected the cuff-electrode to the direct current stimulator (DS2A, Digitimer, Welwyn Garden City, United Kingdom). This setup allowed us to have full control over MTC length and stimulation while measuring GM force (see Fig 3).

#### General procedures.

After securing the rat in the experimental setup, we conducted a series of tetanic contractions for several purposes. First, we performed isometric contractions to ensure that GM pulled in its natural direction. These isometric contractions were also used to determine the supramaximal stimulation current for GM. The stimulation current was gradually increased until GM force plateaued. The current at which no further increase in GM force was observed was then used consistently throughout the experiment. Additionally, the isometric contractions served as a check to confirm that the surgery was successful, particularly ensuring that no GL + PL force was transmitted to the distal GL + PL tendon, and thus that no myofascial force transmission occurred [11]. Subsequently, we performed two to three quick-release, step-ramp and isometric experiments along with two to three SSCs. This approach was based on results from pilot experiments, which indicated that imposing these contractions prior to the start of the experiment resulted in more stable GM force throughout the entire experiment.

Following the preparatory procedures, we commenced the main experiment. The main experiment consisted of two parts. In the first part, we performed quick-release, step-ramp and isometric experiments to estimate the MTC properties of each rat. In the second part, we performed various SSCs. To assess if GM was fatigued, two quick-release and two step-ramp experiments were repeated halfway through the second part of the experiment and again at the end of the entire experiment. Additionally, isometric experiments were performed after every six series of SSCs during the second part of the experiment, using a fixed MTC length 2–4 mm below MTC optimum length. Between contractions, GM was returned to slack length, followed by a two-minute rest period. During these rest periods, GM was irrigated with saline to keep the muscle tissue moist. To reduce potentiation effects, two twitches were applied 1.5 seconds before each contraction.

#### Quick-release, step-ramp and isometric experiments to estimate MTC properties.

Quick-release, step-ramp, and isometric experiments were performed, with the resulting data being used to estimate MTC properties for each rat individually (see Fig. 3 of [12]). This approach served two purposes. First, it enabled us to predict AMPO for the experimentally tested SSCs for each rat individually. As a result, less rats were required to evaluate predictions against experimental results. Second, it enabled us derive predictions of the influence of a broad range of SSC parameters — cycle frequency, FTS and MTC length excursion — on the maximally attainable AMPO for each rat individually. The full experimental procedure of the quick-release, step-ramp and isometric experiments as well as the MTC property estimation has been described in [12], where we demonstrated that these properties can be estimated accurately when accounting for muscle fibre shortening during quick-releases.

#### Stretch–shortening cycles to evaluate predictions.

SSCs were parametrised by cycle frequency, FTS, and MTC length excursion (i.e., maximum minus minimum MTC length, see Fig 2). The average MTC length was set ~ 3 mm below the MTC length yielding maximal isometric force to prevent irreversible damage to the muscle fibres. MTC velocity was constant during shortening and lengthening phases, except around the transition from MTC lengthening to shortening and vice versa. During these transitioning phases, MTC length changed sinusoidally such that MTC length over time was continuously differentiable to the third order. The maximum MTC acceleration during these transitions was limited to 1500 mm/s² to further reduce the risk of irreversible muscle fibre damage. This constraint limited the range of feasible experimental conditions: SSCs with a high cycle frequency, large MTC length excursion, and either a substantially high or low FTS value could not be tested.

**Fig 4:**
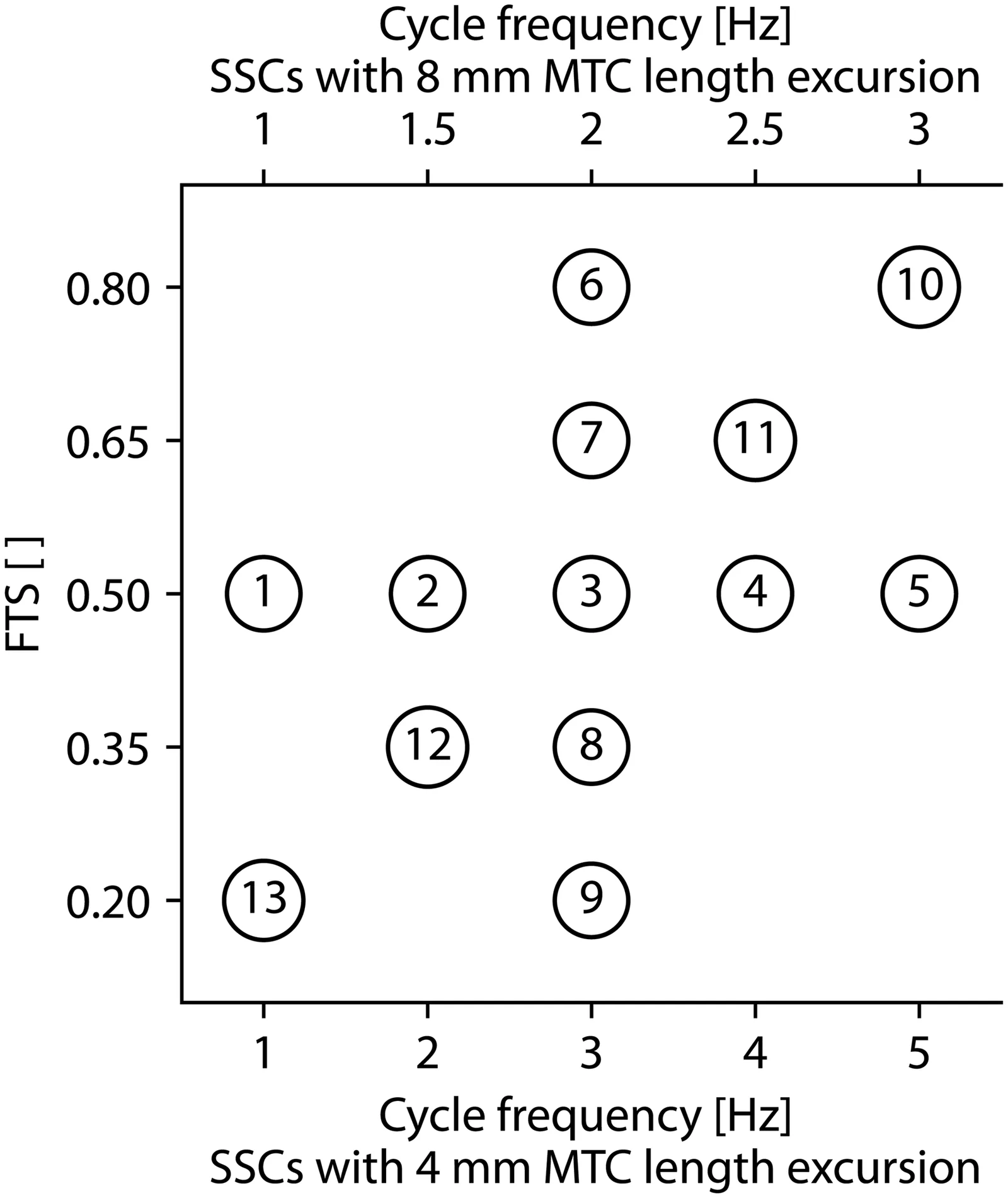
Representation of experimentally investigated SSCs. Thirteen combinations of cycle frequency and FTS were tested at two distinct MTC length excursions (4 mm and 8 mm), resulting in a total of 26 experimental SSC conditions.

Experimental conditions were selected based on preliminary simulations using a Hill-type MTC model with parameter values from literature (see S1 File). We tested 26 conditions, comprising 13 combinations of cycle frequency and FTS (see Fig 4), each at an MTC length excursion of 4 mm and 8 mm. Full details, including muscle stimulation onset time and duration, are provided in the Supporting information (S1 and S2 Tables).

Each SSC condition consisted of five consecutive cycles, with muscle stimulation applied during the middle three cycles. Muscle stimulation onset time was set at the transition from MTC lengthening to shortening. Muscle stimulation duration was based on preliminary model predictions (see S1 File), however, we chose a stimulation duration that was slightly shorter (20 ± 10 ms) than that predicted to be optimal for AMPO. This was done to prevent irreversible damage to the muscle fibres as a possible result of substantial GM force during active GM lengthening. After each SSC condition, we inspected GM force over time to heuristically adjust muscle stimulation duration to further enhance AMPO. The SSC condition was then repeated with the adjusted muscle stimulation duration after all other conditions had been performed for that specific MTC length excursion (~40 minutes later in time). This approach aimed to evaluate to what extent we could enhance AMPO and to evaluate the reliability of measured GM force. Overall, we performed 52 series of SSCs for each rat. For each cycle, AMPO was computed as the product of cycle frequency and the integral of measured GM force over measured MTC length (i.e., the ‘work loop’ area).

Unfortunately, rat 3 passed away near the end of the experiment, which meant that six SSC conditions with an 8 mm MTC length excursion were performed only once instead of twice for this rat, such that 46 instead of 52 experimental SSCs were measured for rat 3.

**Fig 5:**
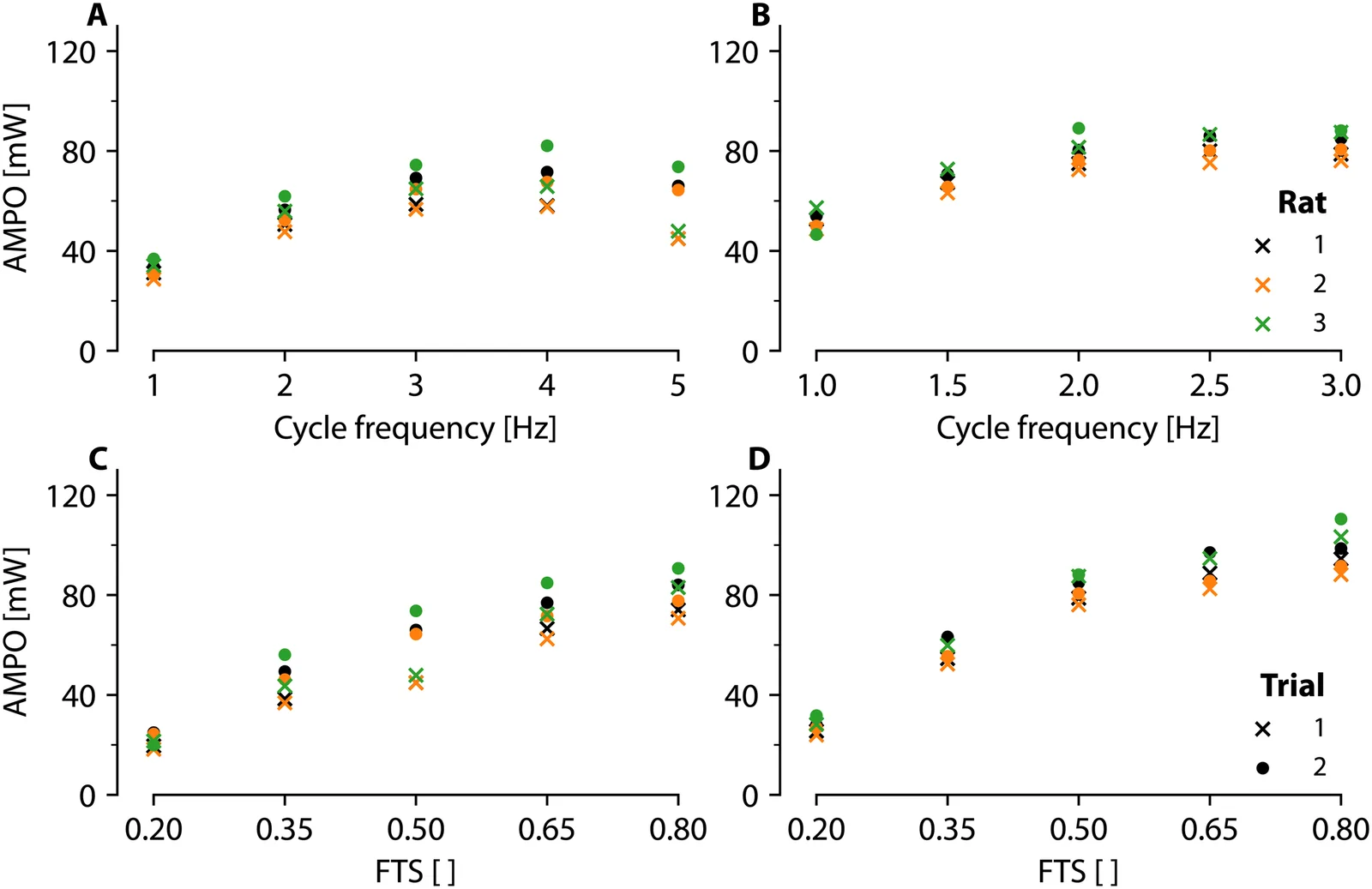
Experimentally measured influence of cycle frequency and FTS on AMPO. AMPO as a function of cycle frequency, with a fixed FTS of 0.5 at an MTC length excursion of 4 mm (**A**) and 8 mm **(B)**. AMPO as a function of FTS, with a fixed cycle frequency of 3 Hz at an MTC length excursion of 4 mm (**C**) and 8 mm **(D)**. Each combination of cycle frequency, FTS and MTC length excursion was performed twice. The first trial with suboptimal muscle stimulation duration and the second trial with an improved muscle stimulation duration.

We tested whether cycle frequency and FTS have a significant effect on the experimentally measured AMPO. This was done on each of the four subsets of the data where one SSC parameter was held constant, which are shown in Fig 5. Thus, in total, four statistical tests were performed (two for cycle frequency and two for FTS at the two MTC length excursions). Effects of cycle frequency and FTS (linear and quadratic) were assessed using a linear mixed-effects model, with cycle frequency or FTS as a fixed effect and specimen as a random effect to account for repeated measurements. No correction was made for differences in stimulation duration, so results can be considered a conservative estimate of the statistical effect of cycle frequency/FTS. Formally, the model can be expressed as:


$$\begin{array}{c} y_{ij}=\mu +a\cdot x_{ij}+b\cdot x_{ij}^{2}+u_{j}+\epsilon_{ij},\;u_{j}\sim N(0,\sigma_{u}^{2}),\;\epsilon_{ij}\sim N(0,\sigma^{2}) \end{array}$$

where $y_{ij}$ is the experimentally measured AMPO for observation *i* in specimen *j*, $x_{ij}$ is the corresponding predictor value, *a* and *b* are the fixed linear and quadratic effects of *x* (cycle frequency or FTS), $u_{j}$ is the random effect for specimen, and $\epsilon_{ij}$ is the residual error.

### MTC model

A Hill-type MTC model was used to represent GM. The model has been described extensively elsewhere [12,13]. In short, the MTC modelled consisted of a contractile element (CE) and a parallel elastic element (PEE) which were both in series with a serial elastic element (SEE). PEE and SEE were modelled as purely passive elastic structures [14], with their force non-linearly dependent on their length. The behaviour of CE was more complex: CE force depended on CE length, CE velocity and active state (*q*), which was defined as the relative amount of *Ca*^2+^ bound to troponin C [15]. The excitation dynamics were modelled according to Hatze [16, pp 31–32]. Here, active state depended on the normalised concentration of free *Ca*^2+^ between the filaments (γ) and CE length, with the sensitivity of active state *q* to *Ca*^2+^ increasing with CE length [e.g., 17, 18]. The normalised concentration of free *Ca*^2+^, in turn, was related to the normalised muscle stimulation (*STIM*) through a first-order differential equation. The independent inputs of the model were *STIM* and MTC length over time, while CE length and γ were the states.

#### Evaluation of measured versus predicted AMPO.

Experimental measurements were compared with predictions derived using a Hill-type MTC model. To ensure a fair comparison, MTC properties were not tuned to the experimental SSC data; instead, they were obtained from dedicated trials see [see [12]]. Moreover, all model inputs were identical to those measured experimentally. Specifically, we simulated all experimental SSC conditions for each rat individually based on their estimated MTC properties, using two inputs: the experimentally measured MTC length over time and *STIM(t)* derived from the measured direct current stimulator signal. The latter was done because the measured direct current stimulator signal consisted of a train of pulses. *STIM* was computed as follows: *STIM* was maximal (i.e., a value of 1) between two stimulation pulses and was zero exactly halfway through the inverse of the stimulation frequency (i.e., 5 ms) after the last stimulation pulse. Model-predicted AMPO was then compared with measured AMPO to evaluate predictive accuracy using the coefficient of determination (r^2^) derived from Pearson’s correlation [19].

#### Imposed stretch–shortening cycles — constant MTC shortening/lengthening velocity.

To systematically explore the influence of SSC parameters on the maximally attainable AMPO, we derived predictions with a Hill-type MTC model based on estimated MTC properties specific to each rat. Specifically, we examined: 1) how the effect of MTC length excursion on the maximally attainable AMPO varied with FTS; 2) how sensitive the maximally attainable AMPO was to changes in SSC parameters and 3) how optimal values of cycle frequency, FTS, and MTC length excursion co-varied in order to maximise AMPO.

During the imposed SSC, MTC shortening and lengthening velocities were set to constant values throughout their respective phases — thus imposing no constraint on MTC acceleration — in order to explore a broad range of cycle frequency, FTS, and length excursion combinations. The maximally attainable AMPO was predicted for 2280 combinations of cycle frequency (0.5–6.0 Hz in 0.5 Hz steps), FTS (0.05–0.95 in 0.05 steps), and MTC length excursion (2–11 mm in 1 mm steps). For each combination and each rat, we optimised the muscle stimulation onset time (i.e., the time instance at which stimulation switches from 0 to its maximal value) and muscle stimulation duration (i.e., the time period during which stimulation remains at its maximal value before returning to 0) to maximise AMPO using a Nelder-Mead simplex method [20].

To ensure periodic behaviour, simulations were run for at least five cycles and continued thereafter until the difference in GM force at the start and end of the cycle was less than 20 mN. To verify that the global optimum had been found, we systematically varied stimulation onset time and duration. No increase in maximally attainable AMPO was observed across any SSC following changes in muscle stimulation.

Finally, as the simulations also served as a comparison with the experimental results of Askew & Marsh [8,9], we reduced the SEE slack length in our simulations. In their setup, a clip was attached to the distal tendon as closely as possible to the muscle fibres, while the proximal tendon was left intact. Based on this, we simulated the SSCs with a reduced SEE slack length of 3 mm for all rats.

#### Imposed stretch–shortening cycles — optimal MTC shortening/lengthening velocity.

The last aim in this study was to identify the MTC length over time for maximal AMPO, when MTC length over time was entirely free to take any form. This allowed us to quantify how much more AMPO could be produced when the SSC shape was completely unconstrained, compared to SSCs with constant MTC shortening and lengthening velocities. In the Hill-type MTC model used, SEE and PEE were assumed to be purely elastic [e.g., [13, 21] and therefore did not contribute to AMPO. Hence, only CE behaviour affected AMPO. The optimisation problem was thus reduced to finding the periodic CE length and stimulation over time in order to maximise AMPO. These optimisation problems were solved with Optimal Control techniques (see S2 File) for imposed cycle frequencies ranging from 0.5 to 6.0 Hz. Out of 615 optimisations, 604 converged successfully. Among these, the difference between maximum AMPO and the submaximal solutions was 0.3 ± 1.1%.

## Results

### *In situ* experiment

#### Reliability of experimental force measurements.

To investigate the influence of SSC parameters on the maximally attainable AMPO of only GM, it was essential to have minimal mechanical interaction between GM and GL + PL. Our setup proved effective: GL + PL force remained below 0.2 N in all rats and conditions, with a maximum within-trial standard deviation of 15 mN. For reference, typical values of maximal isometric force of GL + PL are about 14 N [22], while the maximal isometric force of GM in our study was about 16 N on average. We therefore concluded that the effect of GL + PL on GM was negligible.

Several checks were used to assess the stability of GM force throughout the experiment. During the first part of the experiment (i.e., the quick-release, step-ramp, and isometric protocols), the maximum isometric GM force in the step-ramp experiments exhibited a coefficient of variation below 1%. This same low coefficient of variation (<1%) was also observed for the maximum isometric GM force during the isometric contractions in the second part of the experiment (i.e., during the SSCs). The repeated quick-release experiments revealed that GM force was ~ 11 ± 8% lower midway compared to the start of the experiment, with no change midway compared to the end of the experiment. Maximal isometric GM force did, however, hardly decrease over the entire experiment. This suggests, in line with literature [e.g., 23], that the decrease in GM force was more likely caused by a shift in the MTC force–length relationship, potentially due to a decrease in SEE stiffness, rather than by a decrease in maximal isometric GM force. Based on the repeated quick-release experiments, we estimated the shift of the MTC force–length relationship to be ~ 0.5 ± 0.2 mm midway compared to the start of the experiment, with no change midway compared to the end of the experiment. GM force during the ramp was ~ 5 ± 3% lower midway compared to the start of the experiment, but remained stable in the second part of the experiment. With regard to individual SSC trials, we found that the second- and third-highest experimentally measured AMPO values among the three stimulated cycles were on average only 4  ±  3% lower than the highest value across all conditions and rats. Additionally, GM force development was similar in the first and second trial, which had similar muscle stimulation onset times. As intended, the second trial yielded a higher AMPO (13  ±  11% across all SSC conditions and rats) due to an adjusted stimulation duration. Overall, GM behaviour was stable and reproducible throughout the whole experiment.

#### MTC property estimation.

The estimated MTC properties showed remarkable consistency across all rats (Table 1). Based on the estimated MTC properties, we derived the maximum CE shortening velocity ($v_{CE}^{max}$; see Table 1), the maximum instantaneous CE power output ($P_{CE}^{max}$; see Table 1) and the CE velocity at which $P_{CE}^{max}$ occurs ($v_{CE}^{opt}$; see Table 1). Notably, the time to reach 50% of maximal isometric CE force was relatively long — approximately 34 ms at MTC optimum length in all rats. In addition to delays caused by excitation dynamics, the relatively compliant SEE substantially influenced GM force development. To exclude the influence of SEE, we calculated the time to reach 50% of maximal isometric force while holding CE length constant at its optimal value. The resulting value was considerably shorter (~12 ms vs ~ 34 ms; see Table 1) and provides a better comparison to the half-rise times at constant CE length reported in literature.

**Table 1:** Estimated MTC properties of rat *m. gastorcnemius medialis* as well as metrics derived from these properties.

Description	Symbol	Unit	Rat 1	Rat 2	Rat 3
*MTC properties*					
Hill constant	$a^{rel}$	–	0.569	0.712	0.649
Hill constant	$b^{rel}$	–	5.97	7.15	6.80
maximal isometric CE force	$F_{CE}^{max}$	N	15.5	14.1	17.0
PEE stiffness scaling factor	$k_{PEE}$	N/mm^2^	28.7	24.1	34.8
SEE stiffness scaling factor	$k_{SEE}$	N/mm^2^	952	1255	1050
CE optimum length	$L_{CE}^{opt}$	mm	13.7	14.7	13.3
PEE slack length	$L_{PEE}^{0}$	mm	15.1	15.6	14.5
SEE slack length	$L_{SEE}^{0}$	mm	28.8	29.3	25.2
Calcium dynamics activation time constant	$\tau_{act}$	ms	55.2	57.7	41.6
Calcium dynamics deactivation time constant	$\tau_{deact}$	ms	27.1	25.3	22.4
*Derived metrics*					
MTC length yielding maximal isometric SEE force	$L_{MTC}^{opt}$	mm	46.6	47.3	42.5
maximal instantaneous CE power	$P_{CE}^{max}$	mW	316	319	352
‘half-rise time’	$t_{HRT}$	ms	13.2	13.8	9.97
maximal CE shortening velocity	$v_{CE}^{max}$	mm/s	144	147	140
CE velocity at $P_{CE}^{max}$	$v_{CE}^{opt}$	mm/s	54.1	57.8	53.8

#### Stretch–shortening cycles.

The experimentally measured AMPO of the SSC conditions are depicted in Fig 5 and detailed in S1 and S2 Tables. Both cycle frequency and FTS had a strong effect on the experimentally measured AMPO at each of the two MTC length excursions, with both the linear and quadratic components being highly significant (p ≪ 0.001). For brevity, we refer to an SSC condition with a cycle frequency of 2 Hz and an MTC length excursion of 8 mm as 8 mm@2 Hz.

In the SSC conditions with FTS set to 0.5, experimentally measured AMPO peaked for ~4 mm@4 Hz and for ~8 mm@2.5 Hz (Fig 5). This indicates that the cycle frequency that maximises AMPO depends on the MTC length excursion. Average MTC velocity was 32 mm/s for 4 mm@4 Hz, and 40 mm/s for 8 mm@2.5 Hz. Since GM force was almost zero at the start and end of shortening, average CE shortening equalled average MTC shortening velocity. This shows that average CE velocity that maximises AMPO is substantially lower than the CE velocity at which instantaneous mechanical power output is maximal (~55 mm/s; Table 1).

In the SSC conditions with fixed cycle frequency, experimentally measured AMPO increased with FTS, reaching its highest measured value at the maximum imposed FTS of 0.8 (Fig 5). This relationship was consistent across both 4 mm and 8 mm MTC length excursions, suggesting that the FTS yielding maximal AMPO is largely independent of MTC length excursion. The effect of FTS on AMPO was most pronounced at low cycle frequencies. For example, raising FTS from 0.2 to 0.5 increased AMPO by ~205% for 4 mm@3 Hz and ~185% for 8 mm@2 Hz, while the increase in AMPO from FTS 0.5 to 0.8 was substantially smaller with 20% for both 4 mm@3 Hz and 8 mm@2 Hz (Fig 5). Since the effect of increasing FTS from 0.65 to 0.80 was small — 8% for 4 mm@3 Hz and 4% for 8 mm@2 Hz — we conclude that the FTS yielding maximal AMPO for these combinations of cycle frequency and MTC length excursion was close to 0.8. Lastly, the effect of FTS on AMPO became more pronounced at higher cycle frequencies. For example, increasing FTS from 0.5 to 0.8 increased AMPO by ~50% for 4 mm@5 Hz and ~40% for 8 mm@3 Hz, which is substantially more than the 8% and 4% increase for 4 mm@3 Hz and 8 mm@2 Hz (see S1 and S2 Tables).

### MTC model

#### Evaluation of measured versus predicted maximally attainable AMPO.

We aimed to explore the influence of SSC parameters – cycle frequency, FTS and MTC length excursion – on the maximally attainable AMPO across a broad SSC parameter space. As discussed earlier, such a comprehensive investigation is not feasible experimentally because only a limited set of conditions can be tested. Therefore, we used a Hill-type MTC model to investigate a broad SSC parameter space. For this modelling approach to be meaningful, it was crucial that the model could accurately predict the influence of MTC length and stimulation over time on AMPO.

**Fig 6:**
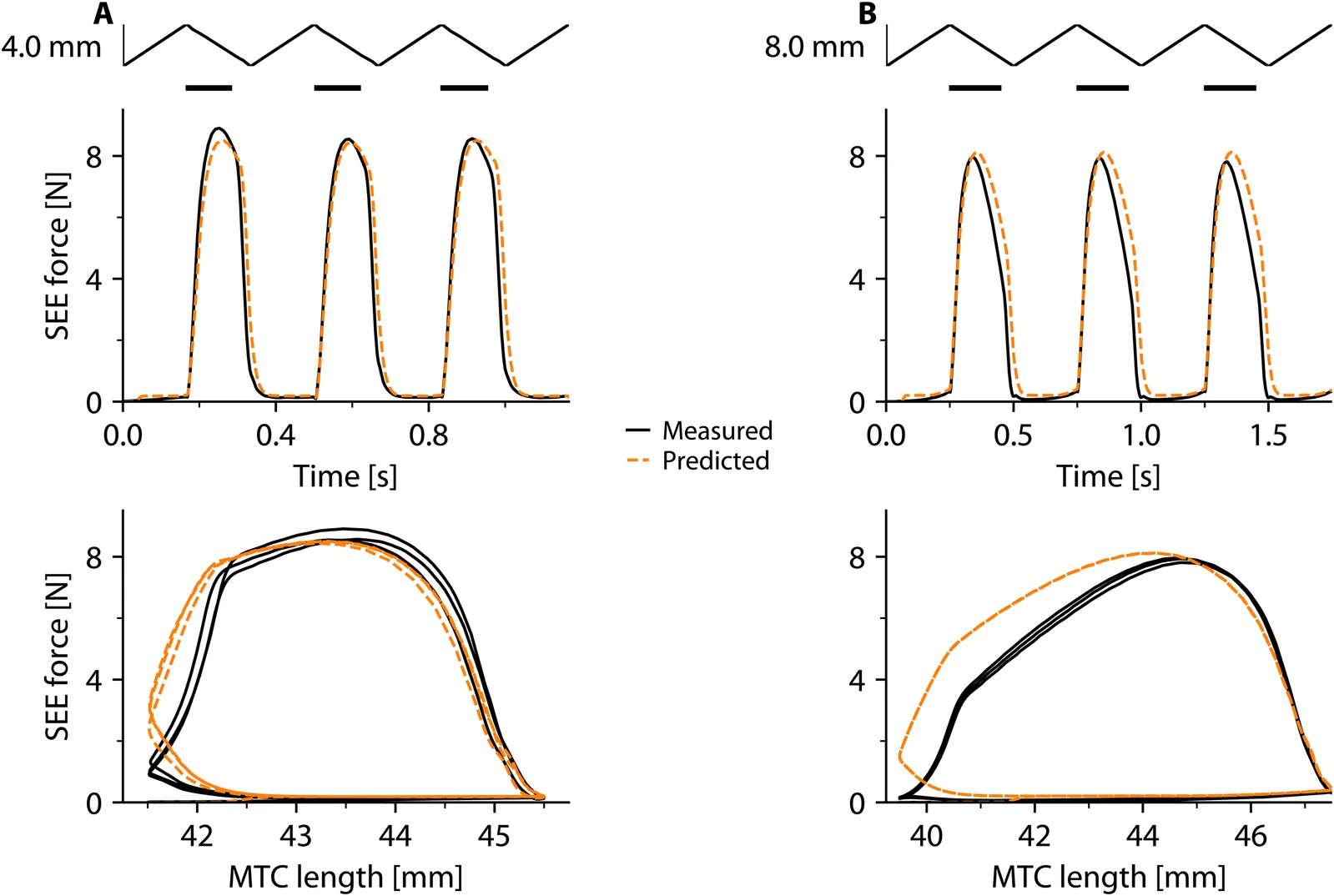
Comparison of experimentally measured and predicted SEE force over time. Predicted SEE force over time was derived using a Hill MTC-type model, with experimentally measured MTC length and stimulation over time as inputs. Predicted SEE force closely matched experimentally measured SEE force during activation but was slightly higher than experimentally measured during relaxation SEE force in most conditions. Top: MTC length over time. Second: Muscle stimulation over time, with maximal stimulation during the periods indicated by the black bars and no stimulation elsewhere. Third: SEE force over time. Bottom: SEE force as a function of MTC length (‘the work loop’). **A)** SSC with a cycle frequency of 3 Hz, an FTS of 0.5 and an MTC length excursion of 4 mm. **B)** SSC with a cycle frequency of 2 Hz, an FTS of 0.5 and an MTC length excursion of 8 mm.

**Fig 7:**
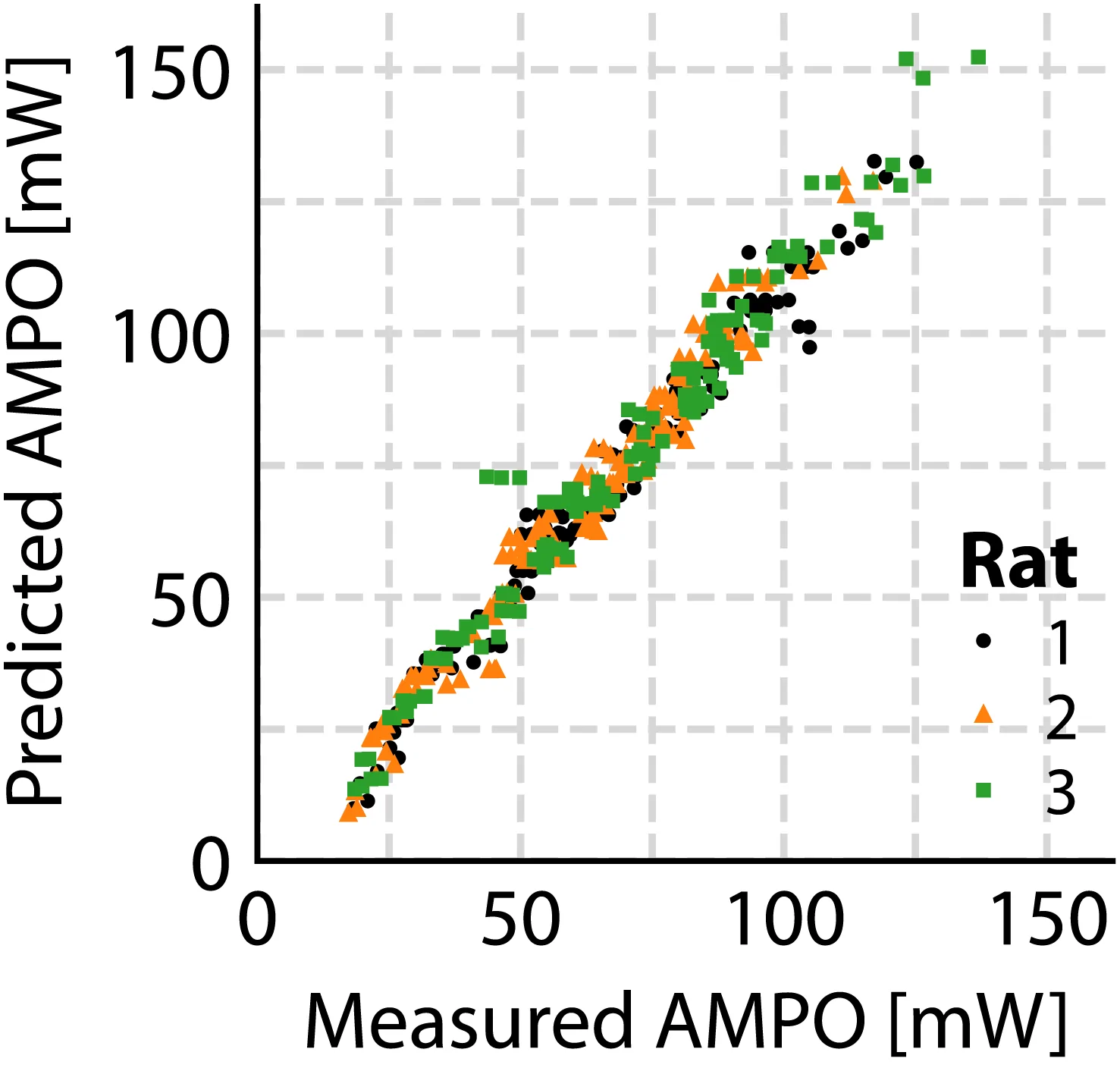
Comparison of experimentally measured and predicted AMPO. Measured AMPO was derived from experimentally observed MTC length and GM force. Predicted AMPO was derived using a Hill MTC-type model, with experimentally measured MTC length and stimulation over time as inputs to predict GM force. Each dot represents AMPO of one full cycle where muscle stimulation was present, such that there are three dots for each experimental SSC condition. The nearly perfect correlation demonstrates that a Hill-type MTC model can accurately predict the influence of MTC length and stimulation over time on AMPO.

The model-predicted GM force closely matched the measured GM force during activation across all rats and SSC conditions (Fig 6). During relaxation, measured GM force was slightly lower than model-predicted GM force in most conditions. As a result, measured AMPO was on average 6  ±  13% lower than predicted. Nonetheless, the correlations between measured and model-predicted AMPO were nearly perfect (r^2^ = 0.99, r^2^ = 0.99 and r^2^ = 0.98 for rat 1, 2 and 3, respectively; Fig 7). This was a remarkable result, as neither the model inputs nor the estimated MTC properties were tuned to experimentally measured SSCs. These findings demonstrate that a Hill-type MTC model can accurately predict the influence of MTC length and stimulation over time on AMPO.

#### Imposed stretch–shortening cycles — constant MTC shortening/lengthening velocity.

**Fig 8:**
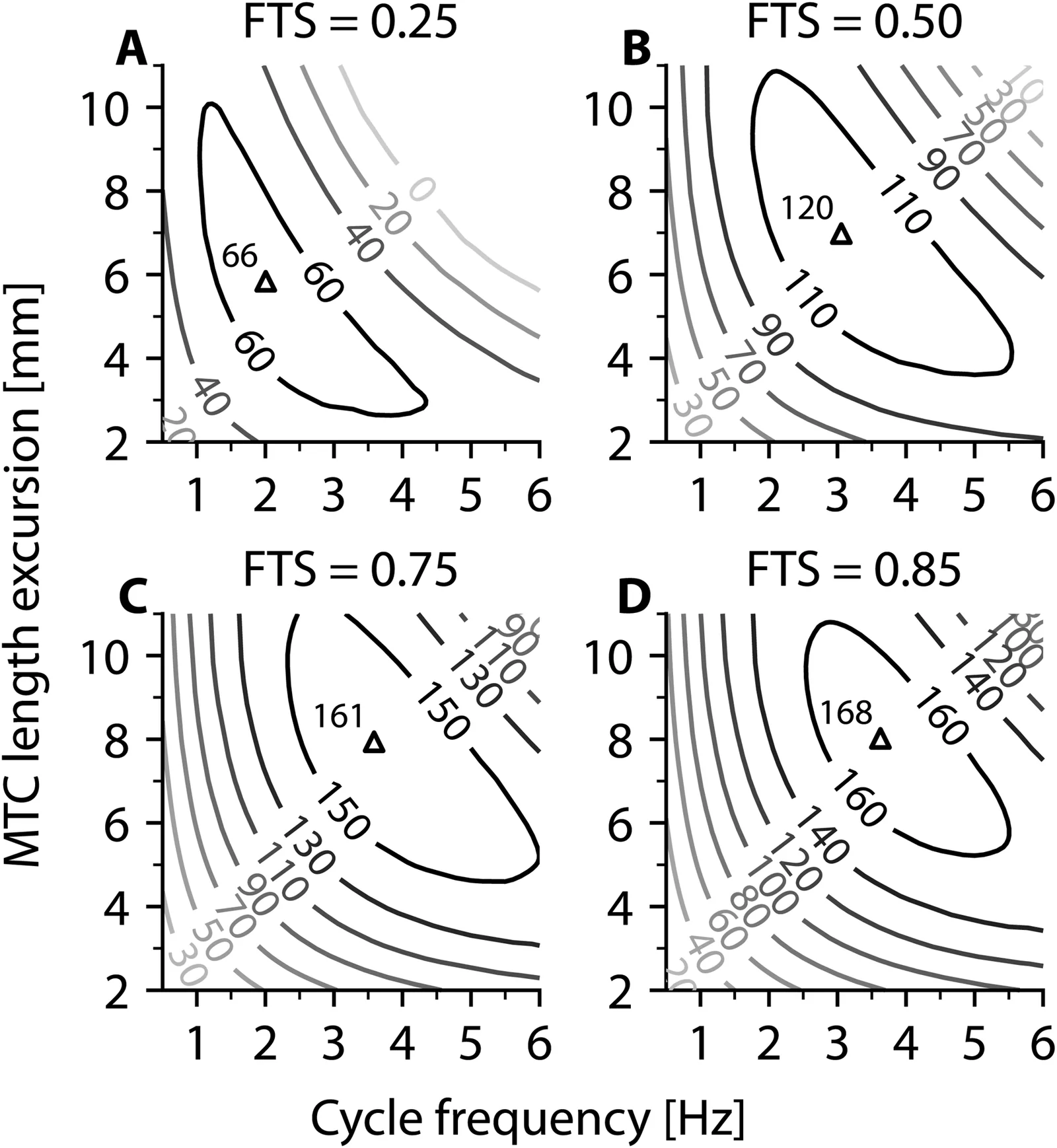
Predicted maximally attainable AMPO as a function of cycle frequency and MTC length excursion, shown for four distinct FTS values. The maximally attainable AMPO (in mW), averaged across three rats, is depicted as contour lines. The open triangles indicate the location of peak AMPO at each FTS corresponding to the optimal combination of cycle frequency and MTC length excursion for each FTS, with the corresponding peak AMPO labelled at the top-left of the triangle.

**Fig 9:**
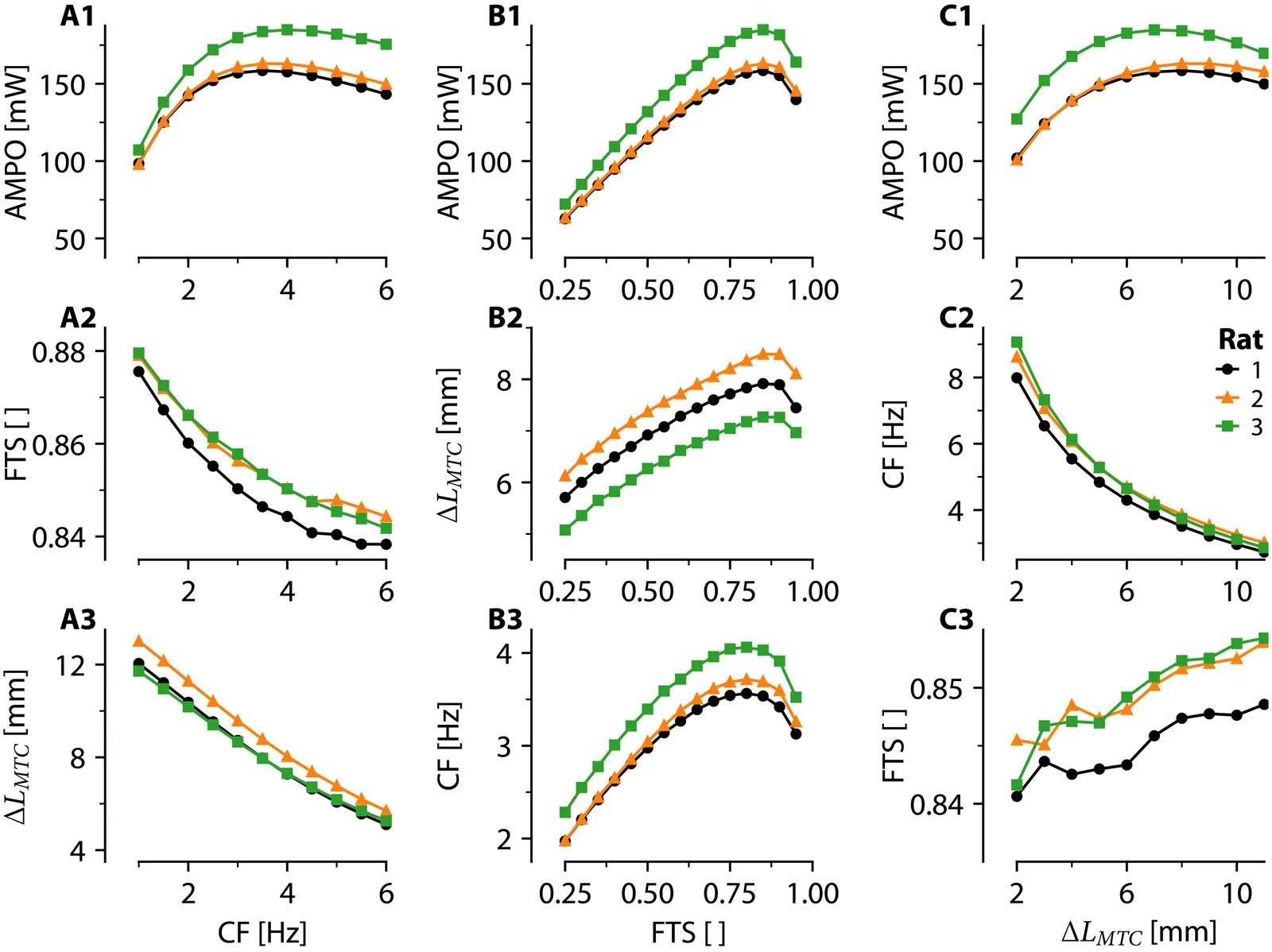
Predicted interrelationships between cycle frequency (CF), FTS, MTC length excursion ($\Delta L_{MTC}$) and the maximally attainable AMPO. These plots collectively illustrate how cycle frequency, FTS, and MTC length excursion interact to maximise AMPO under different conditions. This figure presents a 3x3 grid of plots created by imposing one SSC parameter at a time while optimising the two other SSC parameters to maximise AMPO. In the first column, cycle frequency was imposed, and AMPO (A1) was maximised by identifying the optimal FTS (A2) and MTC length excursion (A3) at each cycle frequency. In the second column, FTS was imposed, and AMPO (A1) was maximised by identifying the optimal MTC length excursion (B2) and cycle frequency (B3) at each FTS. In the third column, MTC length excursion was imposed, and AMPO (C1) was maximised by identifying the optimal cycle frequency (C2) and FTS (C3) at each MTC length excursion. For all columns, muscle stimulation onset time and duration were optimised to maximise AMPO.

Using the Hill-type MTC model, we predicated that AMPO peaks at a value of 169 mW (averaged across the three rats) at cycle frequency of 3.5 Hz, an FTS of 0.85 and an MTC length excursion of 8 mm (Figs 8 and 9). The optimal FTS remained remarkably constant across all tested combinations of cycle frequency and MTC length excursion (Fig 9). By contrast, cycle frequency and MTC length excursion showed a strong interaction, such that when cycle frequency was increased, the optimal MTC length excursion decreased, and vice versa (Fig 9). Notably, a broad range of combinations of SSC parameters achieved AMPO values close to its peak value (Figs 8 and 9). For instance, cycle frequency ranging between 2.9 and 4.6 Hz produced an AMPO within 95% of maximum AMPO, when adjusting FTS and MTC length excursion accordingly. For FTS this was a range between 0.74 and 0.91, when adjusting cycle frequency and MTC length excursion accordingly, while it was between 6.2 and 10.3 mm for the MTC length excursion when adjusting cycle frequency and FTS accordingly. These findings demonstrate that while a clear optimum exists for maximal AMPO, there is a wide variety of combinations of SSC parameters that yield near-maximal AMPO values.

#### Imposed stretch–shortening cycles — optimal MTC shortening/lengthening velocity.

**Fig 10:**
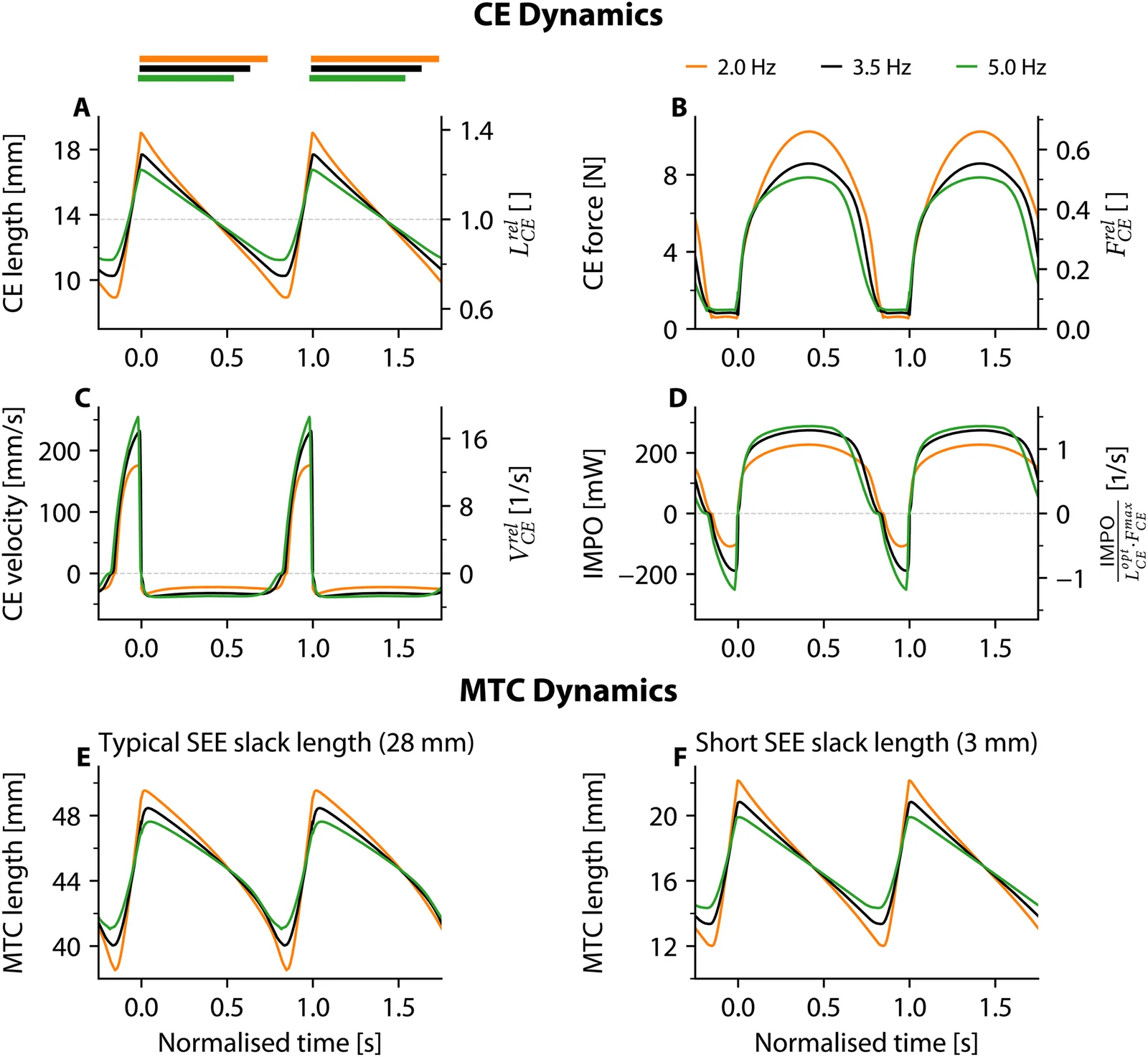
Predicted CE behaviour for maximally attainable AMPO, shown at three distinct cycle frequencies for rat 1. CE length **(A)**, CE force **(B)**, CE velocity (**C**) and instantaneous mechanical power output (IMPO) (**D**) as a function of normalised time (i.e., time divided by the cycle duration). CE stimulation was maximal during the period indicated by the coloured bars and fully off elsewhere. The right labels depict the normalised values, where CE length and CE velocity are normalised to optimum CE length, CE force is normalised to maximal isometric CE force and IMPO is normalised to the product of optimum CE length and maximal isometric CE force. Based on CE length and force over time, and the SEE properties, the MTC length over time can be calculated. The resulting MTC length over time substantially differs between a typical SEE slack length of 28 mm (**E**) and a short SEE slack length of 3 mm **(F)**.

Using the Hill-type MTC model, we identified the optimal CE length over time for maximal AMPO without imposing any constraints on MTC length over time. We found that AMPO was maximised when CE shortened at an approximately constant velocity during the shortening phase and lengthened with increasing velocity during the lengthening phase (see Fig 10), across all imposed cycle frequencies. Given CE length and CE force over time (and PEE and SEE properties), the corresponding MTC length over time can be calculated. Thus, MTC length over time is inherently dependent on the properties of SEE. Because SEE force is not constant during CE shortening, the MTC velocity that maximises AMPO is generally not constant during the shortening phase; even if CE shortening velocity is constant. The size of this effect depends on the SEE slack length: with a typical slack length (e.g., 28 mm), the MTC velocity is clearly not constant (see Fig 10E), whereas with a short slack length (e.g., 3 mm), the MTC velocity closely resembles the CE velocity and is therefore nearly constant during shortening (see Fig 10F).

For a short SEE slack length, the MTC length over time that maximises AMPO has a near-constant shortening and lengthening velocity. As a result, the maximally attainable AMPO using the optimised CE length over time was only 2 ± 1% higher than that achieved with constant MTC shortening and lengthening velocities with a short SEE slack length. It should be noted, however, that this small increase in AMPO is specific to imposing cycle frequency. When imposing FTS or MTC length excursion, the optimal CE length over time was substantially different from that with constant MTC velocities during shortening and lengthening – especially at suboptimal values. For the interested reader, we refer to the Supporting information (S3 File) for the effect of FTS or MTC length excursion on the optimal MTC behaviour for maximising AMPO.

## Discussion

The aim of this study was to explore the influence of SSC parameters — cycle frequency, FTS and MTC length excursion — on the maximally attainable AMPO of rat GM. To this end, we combined *in situ* experiments with simulations and optimisations using a Hill-type MTC model. Model parameter values were estimated individually for each specimen based on independent data from quick-release, step-ramp and isometric experiments. Additionally, the model inputs (i.e., MTC length and stimulation over time) were directly derived from the experimental SSC data. The correlation coefficients between model-predicted and measured AMPO were nearly perfect (r^2^ > 0.98). This is a remarkable result, particularly given that the predictions were not tuned to any of the experimental SSCs. This strong correspondence enabled us to leverage both experimental findings and model-based predictions to investigate how SSC parameters influence the maximally attainable AMPO.

Both experimental data and model-based predictions showed that MTC should spend substantially more time shortening than lengthening in order to maximise AMPO, with an optimal FTS of approximately 0.85. This optimal FTS value remained remarkably constant across all tested combinations of cycle frequency and MTC length excursion. By contrast, cycle frequency and MTC length excursion showed a strong interaction, such that when cycle frequency increased, the optimal MTC length excursion decreased, and vice versa. Lastly, we found that AMPO peaked at a cycle frequency of 3.5 Hz, an FTS of 0.85 and at an MTC length excursion of 8 mm, with a relatively constant CE velocity throughout the shortening phase and an increasing CE lengthening velocity throughout the lengthening phase.

### Robustness and reliability of experimental results

In previous studies, it has been well established that cycle frequency and MTC length excursion significantly affect the maximally attainable AMPO [e.g., 5, 8, 9, 7, 6, 24], whereas the effect of FTS has only been demonstrated by Askew & Marsh [8,9]. Our experimental results also showed significant effects of cycle frequency and FTS on the maximally attainable AMPO, confirming the sensitivity of AMPO to SSC parameters. Understanding how these SSC parameters interact to influence maximally attainable AMPO requires exploring a larger range of muscle lengths and stimulation timings than is experimentally feasible. Therefore, we complemented experiments with simulations and optimisations using a Hill-type MTC model. For this extensive modelling approach into the influence of SSC parameters on maximally attainable AMPO, it was crucial that the model could accurately predict experimental outcomes. Accordingly, our experimental design focused on selecting a representative and informative set of SSCs to evaluate model-based predictions. To assess the reliability of our experimental outcomes, we repeated experimental conditions with similar stimulation timing (three cycles) and with two different timings. The experimental results were highly consistent across the tested specimens. Both the effects of SSC parameters on maximally attainable AMPO (see Fig 5) and the estimated muscle properties (see Table 1) showed minimal inter-individual variation. This aligns with previous findings showing highly reproducible mechanical behaviour in muscles of genetically similar animals, making it unlikely that adding additional animals would change the study outcomes. Furthermore, the effects of SSC parameters on maximally attainable AMPO in our study are in strong agreement with prior findings (see sections below). Taken together, we conclude that our experimental data are robust and provide a reliable basis to evaluate model predictions.

### Optimal FTS for AMPO was relatively constant across all SSC conditions

Our results show that the maximally attainable AMPO substantially increased when FTS increased from 0.25 to 0.50, regardless of cycle frequency and/or MTC length excursion. For example, at the optimal combination of cycle frequency and MTC length excursion, increasing FTS from 0.25 to 0.50 caused AMPO to increase by about 95%. This finding is consistent with the results of Askew & Marsh [8], who reported increases of ~90% for *m. soleus* and ~70% for *m. extensor digitorum longus* when FTS increased from 0.25 to 0.50. Further increasing FTS from 0.50 to 0.75 yielded an additional 30% increase in AMPO, aligning with Askew & Marsh’s observations of a ~ 40% increase in AMPO for both muscles. Moreover, Askew & Marsh [8] observed that AMPO increased even further, and peaked at an FTS between 0.80 and 0.90 at the optimal combination of cycle frequency and MTC length excursion. Our results not only corroborate this finding, but also demonstrate that the optimal FTS was relatively constant (ranging 0.84–0.88) across all tested cycle frequencies (0.5–6 Hz) and MTC length excursions (2–11 mm).

The optimal FTS reflects mainly a trade-off between the CE concentric force–velocity relationship and excitation dynamics. The negative effects of both the CE force–velocity relationship and excitation dynamics on net mechanical work per cycle increase with cycle frequency. Yet, the fact that the optimal FTS remains relatively constant across all cycle frequencies studied suggests that these negative effects increase to a similar extent with cycle frequency (Fig 9). Moreover, because the negative effects of the CE force–velocity relationship and excitation dynamics on AMPO are both strongly and comparably linked to muscle fibre type [25], their combined influence is likely relatively similar across muscles with different fibre type compositions. Consequently, it is perhaps not surprising that the optimal FTS for AMPO remains relatively consistent across different muscles (~0.85 for rat *m. gastrocnemius medialis* in this study; ~ 0.85 for mouse *m. soleus* and *m. extensor digitorum longus* [see [8]] and ~0.80 for human *m. quadriceps femoris* [see 26]). Together, these findings suggest that the optimal FTS is not only relatively constant across all SSC conditions, but also across different species and muscles (with varying fibre types).

### Effect of FTS on optimal cycle frequency and MTC length excursion for maximising AMPO

While the optimal FTS remained relatively constant across all cycle frequencies and MTC length excursions, FTS itself strongly influenced the optimal values of both cycle frequency and MTC length excursion (Fig 9). With respect to cycle frequency, we observed that the optimal value increased with FTS, although the magnitude of this increase diminished at higher FTS values. This observation aligns well with findings by Askew & Marsh [8], who reported comparable increases across FTS values for both *m. soleus* and *m. extensor digitorum longus*. For MTC length excursion, we found that the optimal value increased with 25% and 10% when raising FTS from 0.25 to 0.50 and from 0.50 to 0.75, respectively. A direct comparison with Askew & Marsh [8] is difficult, as their results differed between muscles: for *m. soleus*, these increases were 35% and 40%, while they were 0% and 35% for *m. extensor digitorum longus*. Lastly, Askew & Marsh [8] also examined how average muscle shortening velocity varied with FTS. For *m. extensor digitorum longus*, they found a consistent decrease in optimal shortening velocity as FTS increased. By contrast, *m. soleus* showed a slight increase in optimal shortening velocity, although this discrepancy may stem from missing data near the optimal frequency at FTS = 0.75 (see their Fig. 4 in Askew & Marsh [8]). A follow-up study by the same authors [9] later confirmed that the optimal average shortening velocity for *m. soleus* indeed decreases with increasing FTS, independently of cycle frequency. We observed the same for our model-predictions. Overall, our findings regarding the influence of FTS on optimal cycle frequency, MTC length excursion, and average shortening velocity are in line with previous literature. These findings are not surprising, given the effect of the CE force–velocity relationship. After all, at higher FTS, there is relatively more time for shortening than at lower FTS values. This enables an increase in cycle frequency and allows muscles to shorten and lengthen over a greater distance, without substantial negative effects due to the effect of shortening velocity on muscle force.

### Optimal cycle frequency and optimal MTC length excursion for maximal AMPO

While the optimal FTS for maximal AMPO remained relatively constant across all SSCs studied, we found that cycle frequency and MTC length excursion strongly interacted for maximal AMPO. Specifically, as cycle frequency increased, the optimal MTC length excursion for the maximally attainable AMPO decreased, and vice versa (see Figs 8 and 9). This interaction has previously been observed in sinusoidal SSCs [e.g., 24] – where shortening and lengthening durations are equal. A similar interaction between cycle frequency (i.e., cadence) and crank length—which influences MTC length excursion—has also been observed in human sprint cycling [e.g., 27], where shortening and lengthening durations of individual MTCs are not necessarily equal. However, to our knowledge, this interaction has not been systematically examined in controlled SSCs with systematically varied unequal shortening and lengthening durations; although Askew & Marsh [8] briefly noted this interaction, they did not present the relevant data.

The interaction between cycle frequency and MTC length excursion for maximally attainable AMPO did not come as a surprise, given the negative effect of the CE force–velocity relationship on AMPO. In line with Askew & Marsh [8], Caiozzo & Baldwin [7] and James et al. [24], we observed that the maximally attainable AMPO was less sensitive to increases in cycle frequency and MTC length excursion above their respective optimal values than to decreases below these optimal values. This is not only true at optimal FTS (see Fig 9), but also at suboptimal FTS. The latter can be observed in Fig 8, where the contour lines are more closely spaced below than above the optimum cycle frequency and MTC length excursion. This indicates that, for a given change in cycle frequency or MTC length excursion, AMPO decreases more rapidly below the optimal value than above it. The finding that AMPO was less sensitive to increases in cycle frequency and MTC length excursion above their respective optimal values than to decreases below these optimal values might be counter-intuitive, as one may expect the maximally attainable AMPO to decline rapidly due to a higher shortening velocity (at higher cycle frequencies and MTC length excursion). However, at low cycle frequencies, the MTC must shorten and lengthen over a substantial distance, which reduces the maximally attainable AMPO primarily due the effect of the CE force–length relationship. Conversely, at low MTC length excursions, very high cycle frequencies are required to achieve substantial power output, which in turn compromises AMPO due to the effect of the CE force–velocity relationship (i.e., muscle contracts at lengths at which CE force is substantially lower than the maximal isometric force). In sum, our findings indicate that variations above the optimal cycle frequency and MTC length excursion have a less detrimental effect on the maximally attainable AMPO than variations below optimal values.

### Effect of SEE properties on the MTC behaviour for maximal AMPO

To maximise AMPO, CE should shorten at an approximately constant velocity during the shortening phase and lengthen with increasing velocity during the lengthening phase (see Fig 10). The resulting MTC length over time for maximal AMPO is determined not only by CE length over time, but also by the SEE properties (i.e., SEE compliance and SEE slack length). Due to SEE elasticity, MTC velocity should be higher (i.e., less negative or even positive) than that of CE during force development, and lower (i.e., more negative) during force relaxation in order to maximise AMPO (Fig 10E).

The same principle applies the other way around: when velocity of MTC is constant, that of CE varies during force development and relaxation. Specifically, changes in CE velocity increase with increasing SEE compliance and SEE slack length. In the experimentally observed SSCs, the estimated SEE slack length was substantially longer (~28 mm) than that used in the simulated SSCs (3 mm), resulting in more variable CE velocity during shortening. This variable CE velocity compromises AMPO: for a given shortening distance, CE should ideally shorten at a constant velocity to maximise AMPO. This more variable CE velocity during the shortening phase of the experimental SSC was the main reason why AMPO in the experimental SSCs was lower than in the simulated SSCs. For instance, at a 2 Hz cycle frequency, FTS of 0.5 and an 8 mm MTC length excursion, AMPO in the simulated SSC was ~ 40% higher (~80 mW vs. ~ 110 mW; Fig 5 vs. Fig 8).

We found that – especially at low FTS values, high cycle frequencies and small MTC length excursions – muscle stimulation should occur before MTC shortening starts in order to maximise AMPO. Consequently, CE shortening started before MTC shortening, such that CE was shortening over a longer time interval resulting in a lower average CE shortening velocity than MTC. Thus, the behaviour of SEE does not only cause a discrepancy between instantaneous CE and MTC velocity, it also affects their average shortening velocities.

Askew & Marsh [9] reported that at low FTS values and high cycle frequencies, the average MTC shortening velocity exceeded the CE velocity that maximises instantaneous power output based on the force–velocity relationship ($v_{CE}^{opt}$). They acknowledged that this was a unexpected finding, since CE shortening velocity should theoretically be at or below $v_{CE}^{opt}$ in order to maximise AMPO. Similar to our SSCs with a constant MTC shortening and lengthening velocity, Askew & Marsh [9] left a small portion of the tendon attached to the muscle fibres. Since Askew & Marsh reported that muscle stimulation onset occurred before MTC shortening it is evident that average CE shortening velocity was lower than that of MTC. Therefore, their finding that optimal CE shortening velocity should exceeds $v_{CE}^{opt}$ can, at least, partly be attributed to an overestimation of CE shortening velocity due to the series elastic structures.

The discrepancy between CE and MTC shortening velocity is most pronounced at low FTS, high cycle frequencies, and small MTC length excursion — exactly where Askew & Marsh [9] observed CE shortening velocities exceeding $v_{CE}^{opt}$. At high FTS values, however, this discrepancy is lower. Askew & Marsh [9] found that at high FTS values (i.e., 0.75), the optimal average shortening velocity was consistently below $v_{CE}^{opt}$. This aligns with our findings, even when muscle stimulation onset preceded MTC shortening. In conclusion, while other factors may play a role, we are confident that such factors have little influence on the effect of SSC parameters on the maximally attainable AMPO near the optimum.

### Applications of findings

In most real-world situations, SSCs of individual muscles emerge from the interaction between the musculoskeletal system and the environment. Consequently, the extent to which SSC parameters can be influenced depends on the mechanical context of the task. During flying, for example, birds can and do actively adjust wing kinematics (e.g., by folding and unfolding their wings), thereby enabling a longer shortening than lengthening duration of *m. pectoralis major* [e.g., 28–34]. Our findings help to understand this, as a longer shortening duration is favourable for maximising AMPO. In addition, our results may also shed light on (the evolution of) the “design” of musculoskeletal systems, for example the role of moment-arms which affect MTC length excursion thereby influencing AMPO [e.g., 35].

SSC parameters can be substantially influenced by modifying the interaction between the musculoskeletal system and the environment. One way to achieve this is through the use of external equipment, which can change or constrain movement kinematics. A prime example of such equipment is the bicycle, where crank length affects joint excursions and thus MTC length excursions. Also, non-circular chainrings can be used to vary the gear ratio throughout the crank revolution. This allows the gear ratio to be increased during the downstroke and to be decreased during the upstroke, thereby changing the relative time spent in each phase. In a clever experiment, Martin et al. [36] manipulated chainring shape to alter the relative duration of the downstroke and upstroke during single-leg sprint cycling. AMPO increased with a greater proportion of time spent in the downstroke: an 18% increase was observed when the downstroke occupied 50% rather than 42% of the cycle duration, and a further 4% increase was observed at 58% compared to 50%. Another example is rowing, where the resistance during the stroke and recovery phases could be adjusted in order to alter muscle shortening and lengthening durations. More broadly, such (re)design principles could be applied to any equipment that changes or constrains movement kinematics, thereby enabling the SSCs of the involved muscles to operate closer to their optimum for AMPO.

It should be noted that in motor activities involving multiple muscles (e.g., bicycling, handcycling, rowing, speed skating, etc.), the objective is not to maximise the AMPO of a single muscle. Rather, the goal is to maximise the total AMPO of all muscles combined, which introduces an additional trade-off. In this context, when considering a joint, it should be clear that the optimum FTS for one muscle (e.g., an FTS of ~0.85) cannot be realised in both agonists and antagonists. In this situation, the optimal solution arises from a trade-off that depends on properties of both muscles (e.g., maximal isometric force and muscle fibre length), and is therefore expected to shift in both muscles towards more intermediate values (i.e., closer to an FTS of 0.5). Nevertheless, it stands to reason that larger muscles should operate closer to their individual optimum than smaller muscles in order to maximise performance. Thus, when (re-)designing equipment to maximise total AMPO, configurations should enable larger muscles to operate closer to their individual optimum. Our study provides a starting point for guiding future equipment design aimed at improving total AMPO.

## Supporting information

S1 Table Overview of experimental stretch–shortening cycle conditions with a 4 mm MTC length excursion. The table summarises the SSC parameters, stimulation duration, and measured AMPO for each rat.
(PDF)

S2 Table Overview of experimental stretch–shortening cycle conditions with an 8 mm MTC length excursion. The table summarises the SSC parameters, stimulation duration, and measured AMPO for each rat.
(PDF)

S1 File Selection of experimental SSC conditions. Description of the preliminary Hill-type muscle–tendon complex simulations used to determine the experimental stretch–shortening cycle (SSC) conditions.
(PDF)

S2 File Optimisation protocol to identify optimal MTC length over time. Description of the optimal control problem to find the fully unconstrained MTC length and stimulation over time that maximises AMPO.
(PDF)

S3 File Optimal CE length over time: influence of FTS and MTC length excursion. Detailed results of the predicted optimal MTC length over time for maximising AMPO under imposed FTS values and MTC length excursions.
(PDF)
