# Supplementary material for: Mechanical power output during stretch–shortening cycles of rat medial gastrocnemius muscle: Influence of various muscle length trajectories
Source: PLoS Comput Biol. 2026 Aug 12;22(8):e1014587. doi: 10.1371/journal.pcbi.1014587 (PMC13492780; doi:10.1371/journal.pcbi.1014587)
Supplement: Supplementary file 1 [file pcbi.1014587.s001.pdf]

Table S1: SSC parameters, stimulation durations, and measured AMPO of experimental stretch-shortening cycles with a 4 mm MTC length excursion. Stimulation onset was set at the start of MTC shortening in all conditions.

		Condition												
	**Unit**	**1**	**2**	**3**	**4**	**5**	**6**	**7**	**8**	**9**	**10**	**11**	**12**	**13**
SSC parameters														
Cycle frequency	Hz	1.0	2.0	3.0	4.0	5.0	3.0	3.0	3.0	3.0	5.0	4.0	2.0	1.0
FTS	-	0.50	0.50	0.50	0.50	0.50	0.80	0.65	0.35	0.20	0.80	0.65	0.35	0.20
MTC shortening and lengthening times														
MTC shortening time	ms	500	250	167	125	100	267	217	117	66.7	160	162	175	200
MTC lengthening time	ms	500	250	167	125	100	66.7	117	217	267	40.0	87.5	325	800
Stimulation durations														
Trial 1	ms	405	175	103	65	35	183	133	53	23	85	95	115	135
Trial 2	ms	445[1]	205	123	85	55	213	163	73	33[2]	115	115	135	155[3]
Measured AMPO of Rat 1														
Trial 1	mW	31	51	59	58	45	74	67	38	20	82	74	43	23
Trial 2	mW	34	56	69	72	66	84	77	49	25	104	87	47	27
Measured AMPO of Rat 2														
Trial 1	mW	29	48	57	58	45	71	62	37	18	79	70	40	22
Trial 2	mW	30	52	65	68	64	78	72	46	24	93	79	45	24
Measured AMPO of Rat 3														
Trial 1	mW	34	56	65	66	48	83	72	44	22	91	82	47	25
Trial 2	mW	37	62	74	82	74	91	85	56	20	116	96	54	28

[1] For conditon 1: stimulation duration of rat 3 was 455 ms. [2] For conditon 9: stimulation duration of rat 3 was 23 ms. [3] For conditon 13: stimulation duration of rat 1 was 165 ms.
