# Supplementary material for: Mechanical power output during stretch–shortening cycles of rat medial gastrocnemius muscle: Influence of various muscle length trajectories
Source: PLoS Comput Biol. 2026 Aug 12;22(8):e1014587. doi: 10.1371/journal.pcbi.1014587 (PMC13492780; doi:10.1371/journal.pcbi.1014587)
Supplement: Supplementary file 2 [file pcbi.1014587.s002.pdf]

Table S2: SSC parameters, stimulation durations, and measured AMPO of experimental stretch-shortening cycles with an 8 mm MTC length excursion. Stimulation onset was set at the start of MTC shortening in all conditions.

		Condition												
	**Unit**	**1**	**2**	**3**	**4**	**5**	**6**	**7**	**8**	**9**	**10**	**11**	**12**	**13**
SSC parameters														
Cycle frequency	Hz	1.0	1.5	2.0	2.5	3.0	2.0	2.0	2.0	2.0	3.0	2.5	1.5	1.0
FTS	-	0.50	0.50	0.50	0.50	0.50	0.80	0.65	0.35	0.20	0.80	0.65	0.35	0.20
MTC shortening and lengthening times														
MTC shortening time	ms	500	333	250	200	167	400	325	175	100	267	260	233	200
MTC lengthening time	ms	500	333	250	200	167	100.0	175	325	400	66.7	140	433	800
Stimulation durations														
Trial 1	ms	405	253	175	135	103	295	245	115	55	173	175	163	145
Trial 2	ms	455	293	205	165	123	345	275	135	75[1]	213[2]	225	193	165[3]
Measured AMPO of Rat 1														
Trial 1	mW	53	67	75	80	79	94	89	55	26	113	95	54	34
Trial 2	mW	54	47	80	86	85	99	97	63	28	121	104	62	35
Measured AMPO of Rat 2														
Trial 1	mW	49	63	73	75	76	88	83	52	24	104	88	50	32
Trial 2	mW	50	66	76	80	81	92	86	55	27	113	95	54	33
Measured AMPO of Rat 3														
Trial 1	mW	57	73	81	87	88	103	95	60	28	123	101	60	37
Trial 2	mW	47	-	89	-	88	110	-	-	32	129	-	-	40

[1] For conditon 1: stimulation duration of rat 3 was 455 ms. [2] For conditon 9: stimulation duration of rat 3 was 23 ms. [3] For conditon 13: stimulation duration of rat 1 was 165 ms.
