# Supplementary material for: Mechanical power output during stretch–shortening cycles of rat medial gastrocnemius muscle: Influence of various muscle length trajectories
Source: PLoS Comput Biol. 2026 Aug 12;22(8):e1014587. doi: 10.1371/journal.pcbi.1014587 (PMC13492780; doi:10.1371/journal.pcbi.1014587)
Supplement: Supplementary file 3 [file pcbi.1014587.s003.pdf]

# S1 Selection of experimental SSC conditions

In the experiment, we aimed to measure an informative and representative subset of stretch–shortening cycles (SSCs) to investigate the effect of the SSC parameters — cycle frequency, FTS and MTC length excursion — on the maximally attainable AMPO. Yet, a key question was: Which SSCs should be selected? To make an informed decision, we performed preliminary simulations using a Hill-type MTC model with parameter values from literature for rat *m. gastrocnemius medialis* of three rats (Zandwijk et al., 1996).

We predicted maximal AMPO across various combinations of cycle frequency and FTS at five distinct MTC length excursions (2, 4, 6, 8 and 10 mm), each centred at an average MTC length ~3 mm below the MTC length yielding maximal isometric GM force. Muscle stimulation onset time (i.e., the instance at which stimulation switches from 0 to its maximal value) was set to the start of MTC shortening. Muscle stimulation duration (i.e., the time period during which stimulation remains at its maximal value before returning to 0) was optimised using the Nelder-Mead simplex method (Gao and Han, 2012). To ensure periodic behaviour, optimisations were run for at least five cycles and until the muscle force at the beginning of the cycle deviated less than 20 mN from its value at the end of the cycle. Maximal AMPO was calculated for the last cycle.

We then made contour plots for every MTC length excursion (Figure S1.1), showing the influence of cycle frequency and FTS on maximal AMPO at each MTC length excursion. For the experimental SSC conditions, we limited the range of FTS between 0.2 and 0.8 because values outside this range will yield very short shortening or lengthening durations. Within this FTS range, we observed that AMPO peaked at about 5 and 3 Hz for an MTC length excursion of 4 and 8 mm respectively. Again, to prevent short shortening and lengthening duration, we set the maximum cycle frequencies accordingly. Based on these findings, we selected three subsets of SSC conditions: 1) constant FTS, varying cycle frequency; 2) constant cycle frequency, varying FTS; 3) varying both cycle frequency and FTS. Because there was overlap in the SSC conditions, this yielded 13 unique SSC conditions for each MTC length excursion, such that there were 26 different SSC conditions in total.

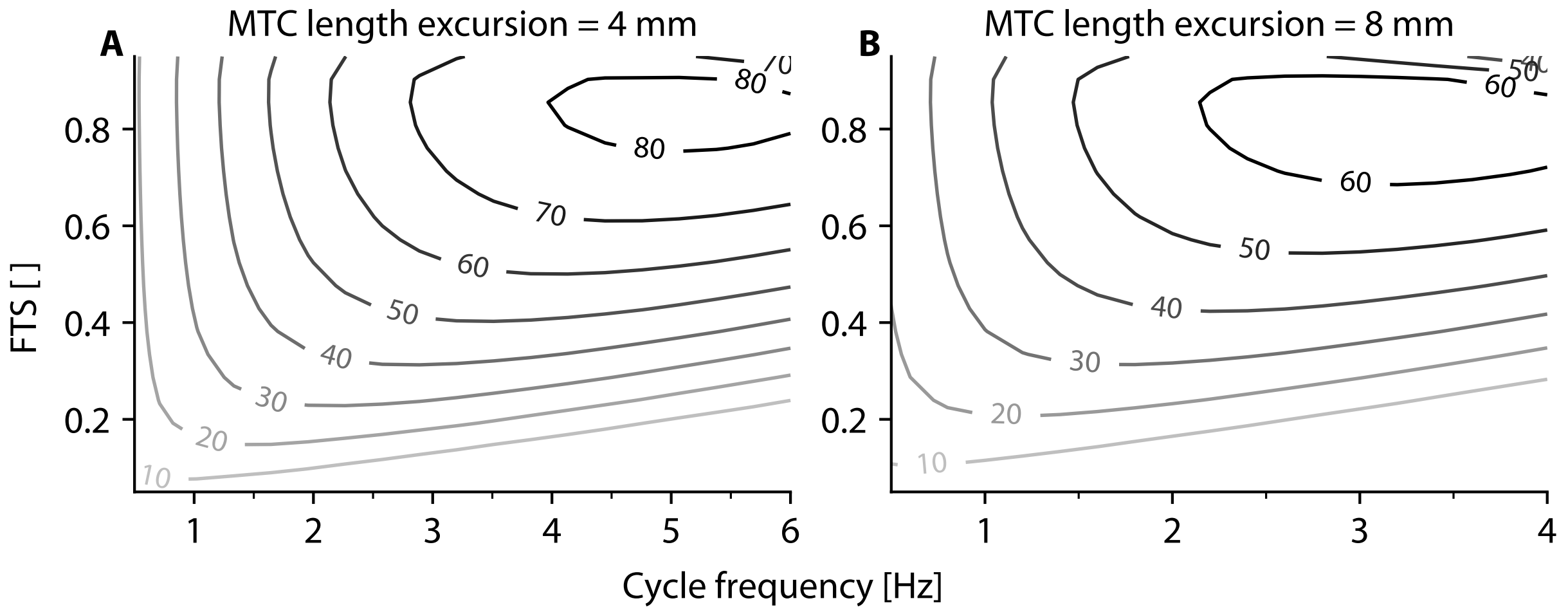


**Figure S1.1: Preliminary predictions of maximal AMPO as a function of cycle frequency and FTS.** The contour lines depict the maximally attainable AMPO (in mW) averaged over three parameter sets. These preliminary predictions were derived with a Hill-type MTC model with parameters obtained from literature (Zandwijk et al., 1996) and were used to inform the selection of experimental SSC conditions.

# References

**Gao, F. and Han, L.** (2012). Implementing the Nelder-Mead simplex algorithm with adaptive parameters. *Computational Optimization and Applications* **51**, 259–277.

**Zandwijk, J. P. van, Bobbert, M. F., Baan, G. C. and Huijing, P. A.** (1996). From twitch to tetanus: Performance of excitation dynamics optimized for a twitch in predicting tetanic muscle forces. *Biological Cybernetics* **75**, 409–417.
