# Supplementary material for: Mechanical power output during stretch–shortening cycles of rat medial gastrocnemius muscle: Influence of various muscle length trajectories
Source: PLoS Comput Biol. 2026 Aug 12;22(8):e1014587. doi: 10.1371/journal.pcbi.1014587 (PMC13492780; doi:10.1371/journal.pcbi.1014587)
Supplement: Supplementary file 4 [file pcbi.1014587.s004.pdf]

S2 Optimisation protocol to identify optimal MTC length over time

One of the aims of this study was to identify the optimal MTC length over time for maximal AMPO, when MTC length over time is entirely free to take any form. To predict this, we used a Hill-type MTC model. In our Hill-type MTC model, we assumed that both SEE and PEE were purely elastic elements (e.g., Anderson and Pandy, 1999; Soest and Bobbert, 1993). Therefore, during fully periodic SSC neither PEE nor SEE contributed to AMPO of the MTC. This also means that neither PEE nor SEE influences the optimal CE length over time that yields maximal AMPO over a full periodic SSC. We used this to simplify the optimisation problem. We reformulated to model such that we considered a Hill-type model of CE only. As such, CE length was no longer a state variable, but an input to the model. The model therefore had two inputs: CE length (L_{CE}) and muscle stimulation ($STIM$) over time. The only state variable was γ, the normalised intrafilament Ca^{2+} concentration. Given this formulation, L_{CE}, $STIM$ and γ were known at each time instant during the optimisation process. Consequently, CE velocity can be calculated by taking the time-derivative of CE length. Using CE velocity, CE force could then be computed using the CE force–velocity relationship. MTC length over time was subsequently obtained by taking the following steps: 1) PEE force was calculated from PEE length (which equals CE length); 2) SEE force was calculated by taking the sum of CE and PEE force; 3) SEE length was calculated from SEE force; 4) MTC length was calculated as the sum of CE and SEE length. By computing MTC length over time via these steps, we could also impose MTC length excursion.

The optimisation problem was thus to find the periodic CE length, $STIM$ and γ over time for maximal AMPO. Optimisations were performed for 1) imposed cycle frequencies (1.0–6.0 Hz in 0.5 Hz steps); 2) imposed FTS values (0.05–0.95 in 0.05 steps) and 3) imposed MTC length excursions (2–11 mm in 1 mm steps). For all optimisation, the following cost function was maximised (note that, in our formulation, CE shortening corresponds to negative CE velocity):

$$J = -\int_{t_0}^{t_0+T_c} F_{CE}^{rel} \cdot v_{CE}^{rel} \, dt \tag{1}$$

where t_0 denotes the initial time and T_c denotes the cycle time.

The solution had to satisfy the differential equation describing the excitation dynamics:

$$\dot{\gamma} = f(\gamma(t), STIM(t)) \tag{2}$$

The following task constraints were added to achieve periodic behaviour:

$$\begin{aligned} L_{CE}(t_0) &= L_{CE}(t_0 + T_c) \\ \gamma(t_0) &= \gamma(t_0 + T_c) \end{aligned} \tag{3}$$

Moreover, inequality constraints were imposed on $\gamma(t)$, $L_{CE}^{rel}(t)$ and $STIM(t)$:

$$\begin{gathered} \gamma_0 \leq \gamma(t) \leq 1 \\ (1-w) \leq L_{CE}^{rel}(t) \leq (1+w) \\ 0 \leq STIM(t) \leq 1 \end{gathered} \tag{4}$$

where L_{CE}^{rel} denotes the CE length normalised to CE optimum length (i.e. the CE length yielding maximal isometric CE force).

We implemented our problem as a Multiphase Optimal Control Problem to assure that CE shortening and lengthening occurred only once per full periodic cycle, which was done by inequality constraints on $v_{CE}^{rel}(t)$:

$$\begin{aligned} v_{CE}^{rel}(t_0 \dots t_0 + T_c \cdot FTS) &\leq 0 \\ v_{CE}^{rel}(t_0 + T_c \cdot FTS \dots t_0 + T_c) &\geq 0 \end{aligned} \tag{5}$$

FTS was a numerical value (between 0.05 and 0.95) when FTS were imposed (see above). When FTS was not imposed, FTS was a control variable optimised for maximal AMPO.

When MTC length excursion was imposed, an extra inequality constraint was added:

$$L_{MTC}(t_0) - L_{MTC}(t_0 + T_c \cdot FTS) = \text{MTC length excursion} \tag{6}$$

The dynamic, task and (in)equality constraints were implemented in CasADi (Andersson et al., 2019) and the cost function was minimised using a Direct Collocation method using a nonlinear interior point method (IPOPT, Wächter and Biegler, 2006). The optimal control solution was checked by running forward simulations of the model with a linear-interpolated $F_{CE}^{rel}(t)$ and $STIM(t)$ as inputs to confirm that no relevant constraints were violated between the collocation points. This was done using SciPy's ODE integrator with an Implicit Runge-Kutta method of Radau IIA family of order 5 (Hairer and Wanner, 1996) and an absolute and relative tolerance of 1e-6 and a maximum timestep of 1e-3. This yielded only very small differences between the states at the collocation points and those at the same time instance of the forward simulation for all optimisations.

References

Anderson, F. C. and Pandy, M. G. (1999). A Dynamic Optimization Solution for Vertical Jumping in Three Dimensions. *Computer Methods in Biomechanics and Biomedical Engineering* **2**, 201–231.

Andersson, J. A. E., Gillis, J., Horn, G., Rawlings, J. B. and Diehl, M. (2019). CasADi: A software framework for nonlinear optimization and optimal control. *Mathematical Programming Computation* **11**, 1–36.

Hairer, E. and Wanner, G. (1996). Implementation of Implicit Runge-Kutta Methods. In *Solving Ordinary Differential Equations II: Stiff and Differential-Algebraic Problems* (ed. Hairer, E.) and Wanner, G.), pp. 118–130. Berlin, Heidelberg: Springer Berlin Heidelberg.

Soest, A. J. van and Bobbert, M. F. (1993). The contribution of muscle properties in the control of explosive movements. *Biological Cybernetics* **69**, 195–204.

Wächter, A. and Biegler, L. T. (2006). On the implementation of an interior-point filter line-search algorithm for large-scale nonlinear programming. *Mathematical Programming* **106**, 25–57.
