# Supplementary material for: Mechanical power output during stretch–shortening cycles of rat medial gastrocnemius muscle: Influence of various muscle length trajectories
Source: PLoS Comput Biol. 2026 Aug 12;22(8):e1014587. doi: 10.1371/journal.pcbi.1014587 (PMC13492780; doi:10.1371/journal.pcbi.1014587)
Supplement: Supplementary file 5 [file pcbi.1014587.s005.pdf]

S3 Optimal CE length over time: influence of FTS and MTC length excursion

Using the Hill-type MTC model, we identified the optimal CE length over time for maximal AMPO without imposing any constraints on MTC length over time. When imposing cycle frequency, the resulting MTC length over time was reasonably similar to that with constant MTC shortening and lengthening velocity. However, when imposing FTS or MTC length excursion the shape of CE and MTC length over time dramatically changed.

For imposed FTS values below the optimal value, CE lengthening velocity was found to be almost zero at the onset of CE lengthening (Figure S3.1A). This infinitely low velocity at the start of CE lengthening was optimal for AMPO because it allowed the active state to decrease fully before lengthening occurred, thereby reducing the amount of negative mechanical work performed. On top of that, it allowed the active state to remain high throughout most part of CE shortening to enable substantial positive mechanical work. Towards the end of CE lengthening, CE velocity again approached zero, which made it possible to initiate muscle stimulation before CE shortening began. This resulted in a high active state at the onset of CE shortening to enhance positive mechanical work. For imposed FTS values above the optimal value, CE shortening velocity decreased over time at the end of CE shortening (Figure S3.1A). To avoid substantial negative mechanical work during the subsequent CE lengthening, muscle stimulation ceased well before the end of CE shortening. While CE could theoretically maintain a constant shortening velocity throughout the shortening phase, any additional positive mechanical work gained near the end was outweighed by the increased negative mechanical work. Thus, an almost isometric phase preceding CE lengthening is optimal for maximising AMPO. In sum, the CE length over time for imposed FTS values mostly results from the effect of the excitation dynamics.

For imposed MTC length excursions above the optimal value, CE shortening velocity was found to be higher at the beginning and end of the shortening phase compared to the middle part (Figure S3.1B). At the start and end of the shortening phase, only limited positive mechanical work could be produced due to the low active state and the suboptimal CE length (that is due to the CE force-length relationship). While CE could theoretically shorten at a constant velocity throughout the entire shortening phase, this would reduce the positive mechanical work during the middle part due to the effect of the force-velocity relationship. At the same time, little additional positive mechanical work could be achieved at the beginning and end of the shortening phase (due to the low active state and suboptimal CE length). Consequently, a non-constant CE shortening velocity — with CE shortening first decreasing and then increasing during the shortening phase — is optimal for maximising AMPO at imposed MTC length excursions above the optimal value. For imposed MTC length excursions below the optimal value, the opposite pattern was found: CE shortening velocity was found to be lower at the beginning and end of the shortening phase compared to the middle part (Figure S3.1B). As explained, only limited positive mechanical work could be produced at the beginning and end of the shortening phase due to the low active state. If CE would shorten with a constant velocity throughout the entire shortening phase, this would decrease the

shortening distance during the middle part (at which the active state is high) and thereby reduces the positive mechanical work. As a result, a non-constant CE shortening velocity — with a slow shortening velocity at the end and beginning of the shortening phase — is optimal for maximising AMPO at imposed MTC length excursions above the optimal value. In sum, the optimal CE length over time for imposed MTC length excursions shows the intricate trade-off between the CE force-length relationship, CE force-velocity relationship and the excitation dynamics.

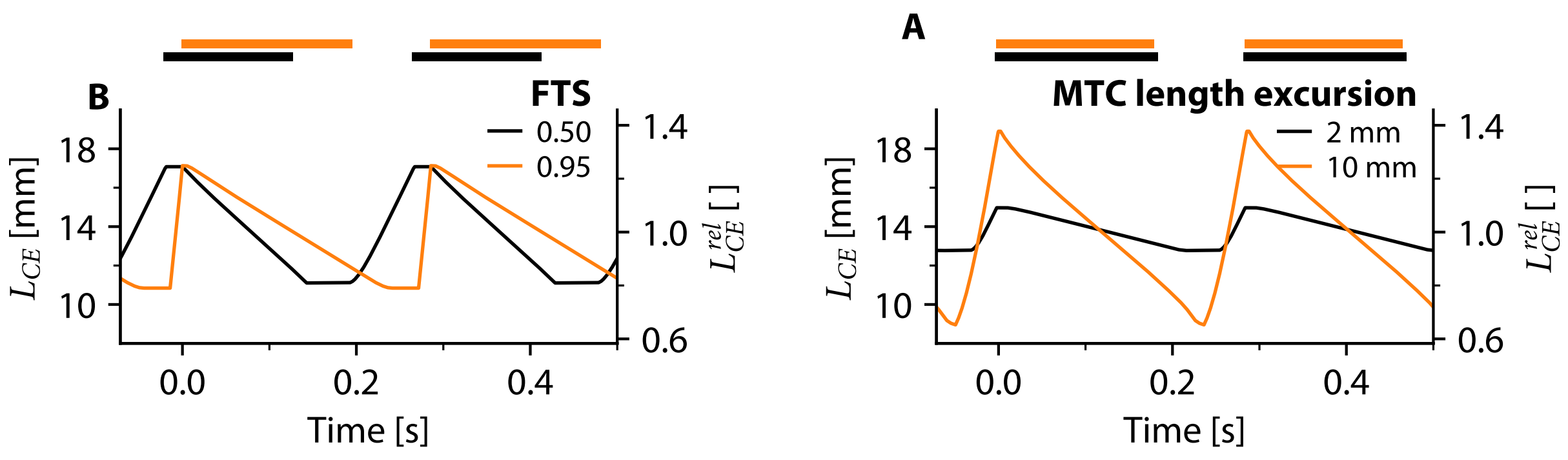

Figure S3.1: Predicted CE length over time for maximally attainable AMPO, shown for two imposed FTS values (A) and two imposed MTC length excursions (B), at a cycle frequency of 3.5 Hz for rat 1. In panel A, only cycle frequency and FTS were imposed, while in panel B only cycle frequency and MTC length excursions were imposed. Apart from these two constraints, CE and MTC length over time were completely unconstrained and thus the shape of CE length over time could be different between every combination of imposed cycle frequency and FTS (A) or imposed cycle frequency and MTC length excursion (B). CE stimulation was maximal during the period indicated by the coloured bars, and 'off' elsewhere.

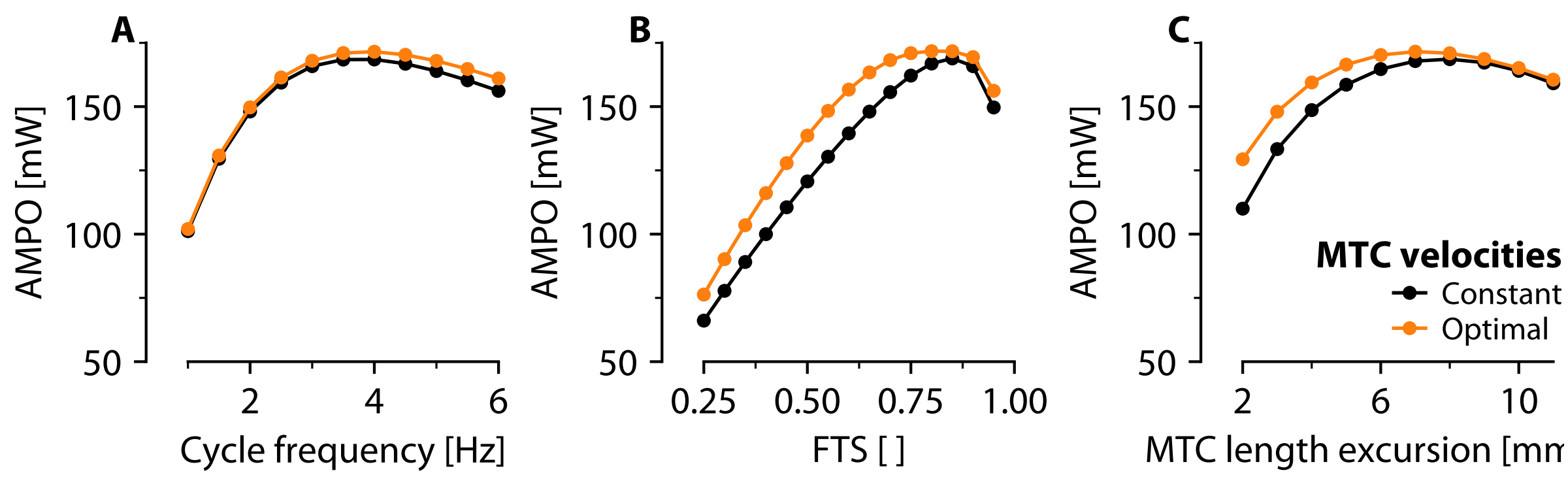

Figure S3.2: Predicted influence of cycle frequency (A), FTS (B) and MTC length excursion (C) on the maximum attainable AMPO. The maximally attainable AMPO — averaged across the three rats — slightly increased in SSCs without a constraint on MTC length over time (orange lines), compared to SSCs with a constant MTC shortening and lengthening velocity (black lines).
