# Supplementary material for: Mechanical power output during stretch–shortening cycles of rat medial gastrocnemius muscle: Influence of various muscle length trajectories
Source: PLoS Comput Biol. 2026 Aug 12;22(8):e1014587. doi: 10.1371/journal.pcbi.1014587 (PMC13492780; doi:10.1371/journal.pcbi.1014587)
Supplement: Supplementary file 6 [file pcbi.1014587.s006.pdf]

Review PCOMPBIOL-D-25-02638, April 6, 2026

SUMMARY

The authors performed muscle contraction experiments on rat medial gastrocnemius, the entire muscle-tendon complex (MTC). Three experimental contraction conditions imposed were used for determining individual parameter values of a Hill-type muscle model, for three muscle specimen. The main focus of interest was to determine the average mechanical power output (AMPO) of a rat MTC over stretch-shortening cycles (SSCs) under varied (non-sinusoidal) cycle conditions, namely with phases of practically constant velocities of stretch and shortening, respectively. The main condition parameters were the relative time spent in shortening (FTS), the cycle frequency, and the MTC's length excursion amplitude; conditions varied and also optimised (in predictive computer simulations of the Hill-type muscle model under the experimental conditions) were the time of stimulation onset at or before shortening, and the length of stimulation time period during the shortening phase. They found that AMPO becomes optimal for high FTS values, at the higher frequencies (3-4Hz) and with larger excursions. AMPO could be even further enhanced (as predicted by the model) if the length trajectory was left free in another optimisation approach, deviating from constant velocities during each stretch and shortening. The impact of varying experimental conditions is discussed in depth and detail. Both the experiments, the modelling and the optimising simulations, and the analysis are entirely sound and have significant scientific value. This is in particular because both approaches have been combined in this study, which is very rarely done in this research field. In addition, the high technical and scientific quality of the study, which roots in decade-long accumulation and transfer of expertise located in Amsterdam, guarantees a substantial contribution to the literature. My (hopefully constructive) criticism addresses only the embedding of the whole approach in literature (of SSCs), which I find a little bit to narrow. Related to this, I think that the motivation (and thus aim) of the study is by far not expressed explicitly enough, accordingly, the discussion lacks something (namely: what is the gain of knowledge about natural biological movement?), and an important issue is not addressed at all, namely, the transfer of the results on AMPO optimisation to muscles (and thus animals) of other sizes. I would strongly encourage to add some discussion on these issues.

REMARKS

1. In the introduction, two (and a half) other studies should be mentioned, or, even better, these studies should be discussed, due to them (1; 2) demonstrating (at least in sinusoidal length oscillations) the impacts of time of activation onset (phase) and, in particular, the length of the STIM period, and other authors (3) estimating, likewise by Hill-type modelling, the effect on work-loops of a titin-like structure, with its activity-history-dependent force contribution in parallel to the cross-bridges. Curtin et al. (1; 2) determined work-loops for fibres only; Kosterina et al. (3) eventually determined likewise fibre-only properties, because tendon elasticity in mice can be expected to be functionally neglectable (much too stiff as compared with rats, cats, piglets; see 100-300g body weight limit for functional significance of tendon material (4, sec. 4.3), (5), (6)), with tendons definitely contributing some dissipation to an MTC's AMPO (7; 8; 9), even though this may be quantitatively neglectable; yet, has anyone calculated this so far? In any case, the phase shift between experimental and model data in your Fig.6 shows, even though pretty subtly, that some friction is missing in the model, be it damping in parallel to SEE or PEE, respectively.

2. I point at these, as I think, substantial studies, because I think you should take them into account, and possibly also discuss them, particularly in the context of this question: For which real-world contraction condition may optimising AMPO be functional? That is, in which biomechanical situation is AMPO a crucial performance criterion? Yes, no question, SSC experiments get closer to examine real-world loading situations than classical steady-state experiments (10). However, it seems that the globally optimal AMPO optimisation strategy as demonstrated in this manuscript (very high FTS = 6/7 = 0.85) is far away from what e.g. humans can realise in natural, non-assisted or -equipped movements as countermovement jumps (seems to be less than 0.5 (11)). Also, in human running (12), FTS is lower than 0.5; and in the extreme case of human long jump (13, fig. 2), the phase of fibre shortening is as low as FTS=0.2; admittedly, steady-state running, long jump, and generally any braking movement (14) do not demand enhancing (forward) speed, i.e. kinetic energy, of the whole body, so AMPO may not be a key criterion that applies. However, optimising AMPO might be expected to be useful in countermovement jumps, as in human breaststroke swimming (15), in which the arms' (flexor muscles) stroke takes a fraction of 7/26 (FTS=0.27) during a whole swimming cycle, and the legs' (extensor muscle) stroke only a fraction of 3/26 (FTS=0.115). I understand that you have dedicated the last part of your discussion (in section 4.6) to externally equipped applications, but it seems to be a fact (at least to me with my limited knowledge about literature) that

AMPO optimisation by such a significant FTS reduction is not within the realm of natural movements, at least in humans; and if I am right, this should be explicitly addressed in the discussion.

3. In regard of my preceding comment, an AMPO value near the optimum (e.g. at 4mm excursion 4Hz with FTS=0.8 at) implies 200ms as the absolute time of shortening, in rats. Could a rat make use of this optimal AMPO performance in all-day life? Could OTHER animals, bigger or smaller (see my comment on scaling below), make use of such globally AMPO-optimal SSCs?

4. If AMPO and its optimisation was primarily chosen to be in the focus of interest because of being a criterion sensitive to dynamic (Hill-type) muscle properties in non-steady-state contraction conditions, and therefore a good indicator for validating a well-established and very useful Hill-type muscle model, then this would be as well a substantial argument in favour of your study. In any case, this motivational and biomechanical context issue should be explicitly addressed in the introduction, and this paper may also significantly benefit from getting back to it in the discussion.

5. Assuming geometric scaling, one could expect that the time scale of fibre contraction would linearly scale with fibre length, and therefore, given the idea that indeed strain rates dominate the AMPO characteristics you have determined, the present AMPO plots (of rats) versus frequency would expectedly be invariant for animals of different size if plotted versus the product of (characteristic movement) frequency, which expectedly scales inversely with time, and optimal fibre length, instead of just frequency; also, AMPO normalised to body mass would expectedly be invariant. May you comment on expected allometry of AMPO characteristics?

WORD CORRECTIONS AND OTHER MINOR COMMENTS

l.130: Remove "data".

l.176: 'used

l.207: I could not unambiguously read from the text whether the excursion (amplitude) is meant peak-to-peak, rather than half that value. Yes, if one has taken a look at $L_{CE,opt}$ values and Fig.2, then it is clear it must be peak-to-peak. Yet, please clarify explicitly here to help the reader: "(peak-to-peak, see Figure 2)".

l.256: Sure you want to say "CE length as sensitivity to Ca^{2+} increases with CE length"? Shouldn't it be "sensitivity of active state q to Ca^{2+} increases with CE length"?

l.257: "The concentration 'of' free Ca^{2+}"

l.265: "based on their" instead of "using on their"

l.312-313: Your F_{CE}^{max} values (sum of GM and GL) are practically identical to what has been given in (16) (30N), as are your animal masses; also, your GMs' F_{CE}^{max} values are consistent with (17) and (18); you may or may not use these sources as validation, just a suggestion.

Table 1: In legend, please, add "of rat GM" after "properties"; I think, it would also be useful to give the three specimens' body mass values in this legend, and, if determined or estimated, muscle mass or volume in the table. Finally, for easy comparison to a lot of other articles on rat muscles, it would be nice to also explicitly present the values of Hill's relative parameters a_{rel} and b_{rel} under "Derived metrics".

Fig.5: In legend, "The first 't'rial with"; I suggest to use the same AMPO range for all sub-panels (0-120mW).

Fig.6: In legend, please, explain that the time of stimulus is depicted by the horizontal bars.

Fig.7: In legend, it should be "Each dot represent's'".

l.380-385: According to what I said at the end of REMARKS 1., you should comment on the phase shift visible in Fig.6.

l.390-392: See REMARKS 5. (scaling).

l.402: "imposing any constraints 'on' MTC length"

l.520-521: "due 'to' a higher shortening"

l.533: SEE slack length and compliance are the only properties modelled, thus, it is enough to write "...SEE properties, its slack length and elastic compliance." [or simply "elasticity"]

l.533-535: As SEE is modelled purely elastic, this sentence just formulates the necessary consequence, not depending on specific SEE parameter values, thus, it should simply write: "Due to SEE elasticity, MTC velocity is always higher (i.e. less negative or even positive) than that of CE during force development (SEE is loaded), and lower (more negative) during force relaxation (SEE is unloaded).".

l.557: Shouldn't it be "before MTC shortening" instead of "before MTC lengthening"?

l.713-714 (also applies to l.299-301): I find this sentence confusing, as the dynamic load-release interaction between CE and SEE (and also PEE, for longer CE lengths) definitely impacts the overall MTC AMPO (10). You ascertain this fact yourself in l.536-550. Still, I understand what you mean, and accordingly I suggest to change the second part of the sentence into "... therefore only the time course of CE, rather than MTC, length changes was AMPO-optimised to simplify the mathematical problem." Moreover, please, add a sentence why minimising the cost function (Eq.(1)) maximises AMPO.

l.718: 'cost function'

l.720-722: Something is confusing here. I try to break it down. If the sentence in l.720 is correct, then Eq.(2) is really ONE equation (rather than a coupled system $\dot{x}(t) = \begin{pmatrix} \dot{\gamma}(t) & \dot{L}_{CE}^{rel}(t) \end{pmatrix}^T$, i.e. on its left side it should simply be '$\dot{\gamma}(t)$', and you should generally remove the symbol 'x' also on its right side, i.e. write '$f(L_{CE}^{rel}(t), \gamma(t), u(t))$'. Moreover, $STIM(t)$ would be the ONLY (control) input to the system, and $F_{CE}^{rel}(t)$, being a function of both states—AND $\dot{L}_{CE}^{rel}(t)$ immediately known from $L_{CE}^{rel}(t)$ always being given as varied by the optimisation—, would rather be an output, i.e. part of the solution to the optimisation problem. That is, formulating the problem as you did, the cost function in Eq.(1) can always be calculated due to $F_{CE}^{rel}(t) = F_{CE}^{rel}(L_{CE}^{rel}(t), \dot{L}_{CE}^{rel}(t), \gamma(t))$

Am I wrong? The alternative would be, that Eq.(2) is really a system of two equations ('$\dot{x}(t)$'), namely contraction and activation dynamics coupled (i.e. the whole Hill-type MTC model), and the inputs would be $STIM(t)$, with $L_{MTC}(t)$ being searched by the optimisation. Is this what you address in l.730 and Eq.(8)?

References

[1] N. A. Curtin, H. L. A. Bartlam-Brooks, T. Y. Hubel, J. C. Lowe, A. R. Gardner-Medwin, E. Bennitt, S. J. Amos, M. Lorenc, T. G. West, A. M. Wilson, Remarkable muscles, remarkable locomotion in desert-dwelling wildebeest, Nature 563 (7731) (2018) 393–396. doi:10.1038/s41586-018-0602-4.

[2] N. A. Curtin, R. C. Woledge, T. G. West, D. Goodwin, R. J. Piercy, A. M. Wilson, Energy turnover in mammalian skeletal muscle in contractions mimicking locomotion: effects of stimulus pattern on work, impulse and energetic cost and efficiency, The Journal of Experimental Biology 222 (Pt 13) (2019) jeb203877. doi:10.1242/jeb.203877.

[3] N. Kosterina, H. Westerblad, A. Eriksson, History effect and timing of force production introduced in a skeletal muscle model, Biomechanics and Modeling in Mechanobiology 11 (7) (2012) 947–957. doi:10.1007/s10237-011-0364-5.

[4] M. Günther, R. Rockenfeller, T. Weihmann, D. F. B. Haeufle, T. Götz, S. Schmitt, Rules of nature's *Formula Run*: Muscle mechanics during late stance is the key to explaining maximum running speed, Journal of Theoretical Biology 523 (2021) 110714. doi:10.1016/j.jtbi.2021.110714.

[5] R. M. Alexander, Tendon elasticity and muscle function, Comparative Biochemistry and Physiology A 133 (4) (2002) 1001–1011. doi:10.1016/s1095-6433(02)00143-5.

[6] A. A. Biewener, R. Blickhan, Kangaroo rat locomotion: Design for elastic storage or acceleration?, The Journal of Experimental Biology 140 (1988) 243–255. doi:10.1242/jeb.140.1.243.

[7] R. F. Ker, Dynamic tensile properties of the plantaris tendon of sheep (*Ovis aries*), The Journal of Experimental Biology 93 (1981) 283–302. doi:10.1242/jeb.93.1.283.

[8] M. Günther, S. Schmitt, V. Wank, High-frequency oscillations as a consequence of neglected serial damping in Hill-type muscle models, Biological Cybernetics 97 (1) (2007) 63–79. doi:10.1007/s00422-007-0160-6.

[9] G. A. Lichtwark, C. J. Barclay, The influence of tendon compliance on muscle power output and efficiency during cyclic contractions, The Journal of Experimental Biology 213 (Pt 5) (2010) 707–714. doi:10.1242/jeb.038026.

[10] N. A. Curtin, C. J. Barclay, The energetics of muscle contractions resembling *in vivo* performance, Journal of Biomechanics 156 (2023) 111665. doi:10.1016/j.jbiomech.2023.111665.

[11] M. Bobbert, K. G. M. Gerritsen, M. C. A. Litjens, A. J. van Soest, Why is countermovement jump height greater than squat jump height?, Medicine & Science in Sports & Exercise 28 (11) (1996) 1402–1412. doi:10.1097/00005768-199611000-00009.

[12] P. V. Komi, Stretch-shortening cycle: a powerful model to study normal and fatigued muscle, Journal of Biomechanics 33 (10) (2000) 1197–1206. doi:10.1016/S0021-9290(00)00064-6.

[13] A. Seyfarth, R. Blickhan, J. L. van Leeuwen, Optimum take-off techniques and muscle design for long jump, The Journal of Experimental Biology 203 (2000) 741–750. doi:10.1242/jeb.203.4.741.

[14] N. Konow, T. J. Roberts, The series elastic shock absorber: tendon elasticity modulates energy dissipation by muscle during burst deceleration, Proceedings of the Royal Society of London B 282 (1804) (2015) 20142800. doi:10.1098/rspb.2014.2800.

[15] A. Bejan, U. Gunes, H. Almahmoud, Locomotion rhythm makes power and speed, Scientific Reports 13 (2023) 14018. doi:10.1038/s41598-023-41023-6.

[16] K. B. Christensen, M. Günther, S. Schmitt, T. Siebert, Strain in shock-loaded skeletal muscle and the time scale of muscular wobbling mass dynamics, Scientific Reports 7 (2017) 13266 (11pp). doi:10.1038/s41598-017-13630-7.

[17] G. J. C. Ettema, P. A. Huijing, Frequency response of rat gastrocnemius medialis in small amplitude vibrations, Journal of Biomechanics 27 (8) (1994) 1015–1022. doi:10.1016/0021-9290(94)90218-6.

[18] C. J. Zuurbier, P. A. Huijing, Changes in geometry of actively shortening unipennate rat gastrocnemius muscle, Journal of Morphology 218 (2) (1993) 167–180. doi:10.1002/jmor.1052180206.
