# Supplementary material for: Mechanical power output during stretch–shortening cycles of rat medial gastrocnemius muscle: Influence of various muscle length trajectories
Source: PLoS Comput Biol. 2026 Aug 12;22(8):e1014587. doi: 10.1371/journal.pcbi.1014587 (PMC13492780; doi:10.1371/journal.pcbi.1014587)
Supplement: Supplementary file 7 [file pcbi.1014587.s007.pdf]

General response

We sincerely thank both reviewers for their careful evaluation of our manuscript. They were highly positive about our experimental work as well as our modelling approach (and the combination thereof). We have used their insightful and constructive comments to further improve the quality of the manuscript. Below, we provide a detailed, point-by-point response to their comments, along with a description of the corresponding revisions made in our manuscript.

Response to reviewer #1

Outline

Comment: *The authors performed muscle contraction experiments on rat medial gastrocnemius, the entire muscle-tendon complex (MTC). Three experimental contraction conditions imposed were used for determining individual parameter values of a Hill-type muscle model, for three muscle specimen. The main focus of interest was to determine the average mechanical power output (AMPO) of a rat MTC over stretch-shortening cycles (SSCs) under varied (non-sinusoidal) cycle conditions, namely with phases of practically constant velocities of stretch and shortening, respectively. The main condition parameters were the relative time spent in shortening (FTS), the cycle frequency, and the MTC's length excursion amplitude; conditions varied and also optimised (in predictive computer simulations of the Hill-type muscle model under the experimental conditions) were the time of stimulation onset at or before shortening, and the length of stimulation time period during the shortening phase. They found that AMPO becomes optimal for high FTS values, at the higher frequencies (3-4Hz) and with larger excursions. AMPO could be even further enhanced (as predicted by the model) if the length trajectory was left free in another optimisation approach, deviating from constant velocities during each stretch and shortening. The impact of varying experimental conditions is discussed in depth and detail. Both the experiments, the modelling and the optimising simulations, and the analysis are entirely sound and have significant scientific value. This is in particular because both approaches have been combined in this study, which is very rarely done in this research field. In addition, the high technical and scientific quality of the study, which roots in decade-long accumulation and transfer of expertise located in Amsterdam, guarantees a substantial contribution to the literature. My (hopefully constructive) criticism addresses only the embedding of the whole approach in literature (of SSCs), which I find a little bit to narrow. Related to this, I think that the motivation (and thus aim) of the study is by far not expressed explicitly enough, accordingly, the discussion lacks something (namely: what is the gain of knowledge about natural biological movement?), and an important issue is not addressed at all, namely, the transfer of the results on AMPO optimisation to muscles (and thus animals) of other sizes. I would strongly encourage to add some discussion on these issues.*

Response: We thank the reviewer for the appraisal of our work and the very valuable and insightful suggestions. After carefully considering both the general outline and specific comments addressed below, we realised that the original manuscript indeed did not sufficiently clarify the primary aim of our study. In particular, we would like to emphasise that our goal was not to investigate how to maximise/optimise AMPO per se. Rather, our aim was much more general: to examine how SSC parameters influence the maximally attainable AMPO. We recognise that the first paragraph of the

Introduction as well as the Title of our ms may have unintentionally misled the reader regarding the primary focus of the study.

We believe that gaining insight in the influence of SSC parameters on the maximally attainable AMPO may give rise to understanding key mechanisms in biological movement and (evolution of) functional anatomy (e.g. moment arms which affect MTC length excursion during movement). It thus helps to understand the trade-off between several aspects of movement, for example the influence of stride frequency and step length on AMPO in running. Obviously, we agree these SSC parameters cannot be fully chosen by the individual. They arise as the results of musculoskeletal properties, neural control and (mechanical) interaction with the environment. This is in particular true for FTS. Our study may provide insight into movement coordination and the underlying trade-offs among the SCC parameters that influence the maximally attainable AMPO.

We have revised our ms (and the title thereof) accordingly to improve the clarity of the study's motivation and aims, and we believe these revisions substantially strengthen the paper. In fact, these clarifications address many of the reviewer's specific concerns outlined below, which we respond to in detail together with the corresponding revisions made to the manuscript.

Remarks

Comment: *1. In the introduction, two (and a half) other studies should be mentioned, or, even better, these studies should be discussed, due to them (1; 2) demonstrating (at least in sinusoidal length oscillations) the impacts of time of activation onset (phase) and, in particular, the length of the STIM period, and other authors (3) estimating, likewise by Hill-type modelling, the effect on work-loops of a titin-like structure, with its activity-history-dependent force contribution in parallel to the cross-bridges. Curtin et al. (1; 2) determined work-loops for fibres only; Kosterina et al. (3) eventually determined likewise fibre-only properties, because tendon elasticity in mice can be expected to be functionally neglectable (much too stiff as compared with rats, cats, piglets; see 100-300g body weight limit for functional significance of tendon material (4, sec. 4.3), (5), (6)), with tendons definitely contributing some dissipation to an MTC's AMPO (7; 8; 9), even though this may be quantitatively neglectable; yet, has anyone calculated this so far? In any case, the phase shift between experimental and model data in your Fig.6 shows, even though pretty subtly, that some friction is missing in the model, be it damping in parallel to SEE or PEE, respectively.*

Response/Action: In this comment, we identify two distinct points, which we address below.

First, we agree with the reviewer that stimulation timing (i.e., onset and duration) influences the net mechanical work per cycle and, consequently, AMPO. In the present study, however, our primary aim was to examine the influence of stretch–shortening cycle (SSC) parameters (specifically cycle frequency, FTS and MTC length excursion) on the maximally attainable AMPO of rat m. gastrocnemius medialis. To isolate the effects of these SSC parameters, stimulation onset and duration were optimised for each combination of SSC parameters. We agree with the reviewer that stimulation timing influences AMPO and that this should be clear for the reader. We have therefore included the references suggested by the reviewer and added the following text in the Introduction:

Line 40-43 in the revised ms: "Since muscle stimulation substantially affects the mechanical behaviour of muscles (e.g. Curtin et al., 2018; Curtin et al., 2019; Sawicki et al., 2015; Stevens, 1996), stimulation onset time and duration are typically optimised to maximise mechanical work

per cycle. This allows isolation of the effects of cycle frequency and MTC/muscle fibre length excursion, and their interaction. Such an approach has been used in experiments to systematically investigate both the individual effects of cycle frequency and/or MTC/muscle fibre length excursion and their interaction on AMPO."

Also, we have added text to clarify that in our study stimulation timing was optimised for each SSC:

Line 109-111 in the revised ms: "Thus, we considered the maximally attainable AMPO for each combination of SSC parameters. To determine this, muscle stimulation was optimised for every SSC."

Second, the reviewer addresses the "phase shift" between experimental and model data in Fig. 6. In Fig. 6 we observed that during activation, the model-predicted m. gastrocnemius medialis (GM) force rise closely matches the experimentally measured force rise. During relaxation, however, the model predicts a slower decline in GM force than observed experimentally. We do not interpret this discrepancy as a phase shift, but rather as a difference during relaxation between model-predicted and experimentally measured GM force.

As suggested by the reviewer, this discrepancy may be related to damping effects and in that light the reviewer noted tendon material is not purely elastic and may contribute to energy dissipation within an MTC's AMPO. In our model, indeed all series connective tissue was represented by a purely elastic series elastic element (SEE), which simplifies physiological reality since tendons and aponeuroses exhibit some viscoelastic behaviour (Ettema & Huijing, 1990). More generally, differences between model-predicted and experimentally measured GM force can arise from any mechanisms not included in the model and/or from imperfect parameter estimation. Given the design of our study, we are not able to identify the exact underlying cause. Addressing this in detail is also beyond the scope of the present manuscript. Nevertheless, the model predictions agreed very well with the experimental data ($r^2 > 0.98$), such that substantial viscous effects in the tendon are unlikely, at least within the context of this study.

Ettema, G.J.C., Huijing, P.A. (1990). Architecture and Elastic Properties of the Series Elastic Element of Muscle-Tendon Complex. In: Winters, J.M., Woo, S.LY. (eds) Multiple Muscle Systems. Springer, New York, NY. https://doi.org/10.1007/978-1-4613-9030-5_4

Comment: 2. *I point at these, as I think, substantial studies, because I think you should take them into account, and possibly also discuss them, particularly in the context of this question: For which real-world contraction condition may optimising AMPO be functional? That is, in which biomechanical situation is AMPO a crucial performance criterion? Yes, no question, SSC experiments get closer to examine real-world loading situations than classical steady-state experiments (10). However, it seems that the globally optimal AMPO optimisation strategy as demonstrated in this manuscript (very high FTS = 6/7 = 0.85) is far away from what e.g. humans can realise in natural, non-assisted or -equipped movements as countermovement jumps (seems to be less than 0.5 (11)). Also, in human running (12), FTS is lower than 0.5; and in the extreme case of human long jump (13, fig. 2), the phase of fibre shortening is as low as FTS=0.2; admittedly, steady-state running, long jump, and generally any braking movement (14) do not demand enhancing (forward) speed, i.e. kinetic energy, of the whole body, so AMPO may not be a key criterion that applies. However, optimising AMPO might be expected to be useful in*

countermovement jumps, as in human breaststroke swimming (15), in which the arms' (flexor muscles) stroke takes a fraction of 7/26 (FTS=0.27) during a whole swimming cycle, and the legs' (extensor muscle) stroke only a fraction of 3/26 (FTS=0.115). I understand that you have dedicated the last part of your discussion (in section 4.6) to externally equipped applications, but it seems to be a fact (at least to me with my limited knowledge about literature) that AMPO optimisation by such a significant FTS reduction is not within the realm of natural movements, at least in humans; and if I am right, this should be explicitly addressed in the discussion.

Response: We agree that the globally optimal AMPO strategy identified in our study (i.e. FTS ≈ 0.85) may not be directly realised in many human movement tasks. In the tasks described by the reviewer the human musculoskeletal system interacts with the environment, such that the SSCs of the muscles arise from muscle forces and mechanical interaction with the environment. Consequently, FTS can only minimally be changed by the individual (e.g., by changing step frequency and/or step length during running).

However, the fact that globally optimal SSC conditions may not be achieved in many real-world movements does not reduce the relevance of the present findings. By quantifying how SSC parameters affect the maximally attainable AMPO, we can, as mentioned before, understand the trade-off between these parameters for AMPO. These insights might help to inform the (re-)design of sport/rehabilitation equipment. In addition, these insights might explain the behaviour observed in certain animals. For example, birds actively modulate wing kinematics (e.g. through partial folding and unfolding), which alters the relative duration of muscle shortening and lengthening phases of the m. pectoralis major, thereby influencing FTS and AMPO.

It should be noted that, in whole-body tasks that involve multiple muscles, the coordination problem is to find muscle activations such that the summed AMPO over all muscles combined is maximised over a full cycle. This inevitably leads to a trade-off between the contributions of individual muscles to the total AMPO. Obviously, increasing the FTS of the extensor muscles would necessarily reduce the FTS of the flexor muscles. Consequently, an FTS value that may be optimal for a single muscle (e.g. FTS ≈ 0.85) is unlikely to be optimal when considering the summed AMPO across all muscles involved. This raises a more general point: if the aim is to maximise the summed AMPO over all muscles, this inevitably introduces a trade-off between individual muscle contributions to the summed AMPO over all muscles. Our results may provide insight into the mechanics underlying such trade-offs.

Action: We first revised our ms in two places.

First, we revised the first paragraph in the Introduction to clarify that our goal was to examine the influence of SSC parameters on the maximally attainable AMPO, rather than to find the global optimum combination SSC parameters for maximising AMPO. The first paragraph now reads:

"In motor activities such as cycling, rowing, and speed skating, muscles periodically shorten and lengthen, a pattern commonly referred to as the stretch–shortening cycle (SSC). When muscles produce force while shortening, they deliver positive mechanical power, and when they produce force while lengthening, they deliver negative mechanical power. During motor activities, part of the mechanical energy delivered by the muscles is dissipated to the environment, and this energy loss generally increases with movement speed. Consequently, performance critically depends on the average mechanical power output (AMPO) that muscles can produce over these SSCs. This raises several fundamental questions: how does muscle length over time influence the maximally

attainable AMPO? More specifically, what are the effects of cycle frequency and muscle length excursion? Which fraction of the cycle should be spent shortening? Do these factors interact, and if so, how? Surprisingly, despite the importance of AMPO for movement performance, many of these questions have not yet been answered."

Second, we revised the section "Application of findings" in the Discussion to better highlight the insights obtained from our study. This section now reads:

"In most real-world situations, SSCs of individual muscles emerge from the interaction between the musculoskeletal system and the environment. Consequently, the extent to which SSC parameters can be influenced depends on the mechanical context of the task. During flying, for example, birds can and do actively adjust wing kinematics (e.g. by folding and unfolding their wings), thereby enabling a longer shortening than lengthening duration of m. pectoralis major [e.g., 27, 28, 29, 30, 31, 32, 33]. Our findings help to understand this, as a longer shortening duration is favourable for maximising AMPO. In addition, our results may also shed light on (the evolution of) the "design" of musculoskeletal systems, for example the role of moment-arms which affect MTC length excursion thereby influencing AMPO [e.g. 36]

SSC parameters can be substantially influenced by modifying the interaction between the musculoskeletal system and the environment. One way to achieve this is through the use of external equipment, which can change or constrain movement kinematics. A prime example of such equipment is the bicycle, where crank length affects joint excursions and thus MTC length excursions. Also, non-circular chainrings can be used to vary the gear ratio throughout the crank revolution. This allows the gear ratio to be increased during the downstroke and to be decreased during the upstroke, thereby changing the relative time spent in each phase. In a clever experiment, Martin et al. [35] manipulated chainring shape to alter the relative duration of the downstroke and upstroke during single-leg sprint cycling. AMPO increased with a greater proportion of time spent in the downstroke: an 18% increase was observed when the downstroke occupied 50% rather than 42% of the cycle duration, and a further 4% increase was observed at 58% compared to 50%. Another example is rowing, where the resistance during the stroke and recovery phases could be adjusted in order to alter muscle shortening and lengthening durations. More broadly, such (re)design principles could be applied to any equipment that changes or constrains movement kinematics, thereby enabling the SSCs of the involved muscles to operate closer to their optimum for AMPO.

It should be noted that in motor activities involving multiple muscles (e.g., bicycling, handcycling, rowing, speed skating, etc.), the objective is not to maximise the AMPO of a single muscle. Rather, the goal is to maximise the total AMPO of all muscles combined, which introduces an additional trade-off. In this context, when considering a joint, it should be clear that the optimum FTS for one muscle (e.g. an FTS of ~0.85) cannot be realised in both agonists and antagonists. In this situation, the optimal solution arises from a trade-off that depends on properties of both muscles (e.g. maximal isometric force and muscle fibre length), and is therefore expected to shift in both muscles towards more intermediate values (i.e. closer to an FTS of 0.5). Nevertheless, it stands to reason that larger muscles should operate closer to their individual optimum than smaller muscles in order to maximise performance. Thus, when (re-)designing equipment to maximise total AMPO, configurations should enable larger muscles to operate closer to their individual optimum. Our study provides a starting point for guiding future equipment design aimed at improving total AMPO."

Comment: 3. *In regard of my preceding comment, an AMPO value near the optimum (e.g. at 4mm excursion 4Hz with FTS=0.8 at) implies 200ms as the absolute time of shortening, in rats. Could a rat make use of this optimal AMPO performance in all-day life? Could OTHER animals, bigger or smaller (see my comment on scaling below), make use of such globally AMPO-optimal SSCs?*

Response: We think that this comment touches on the more general issue addressed before and discussed in #2, namely that we were not only interested in maximising/optimising AMPO, but rather to examine the influence of SSC parameters on the maximally attainable AMPO. Thus, even if animals/humans may not operate under globally optimal combinations of SSC parameters during locomotor tasks, our findings still provide insight into the mechanisms underlying movement behaviour.

Action: We revised the first paragraph of the Introduction as well as the section "Application of findings" in the Discussion. Please see above and/or the revised ms for the texts.

Comment: *4. If AMPO and its optimisation was primarily chosen to be in the focus of interest because of being a criterion sensitive to dynamic (Hill-type) muscle properties in non-steady-state contraction conditions, and therefore a good indicator for validating a well-established and very useful Hill-type muscle model, then this would be as well a substantial argument in favour of your study. In any case, this motivational and biomechanical context issue should be explicitly addressed in the introduction, and this paper may also significantly benefit from getting back to it in the discussion.*

Response: We would argue that the ability to generate substantial AMPO is important in many cyclic tasks. As mentioned earlier, the motivation for our study was that the influence of SSC parameters on the maximally attainable AMPO remains unclear. Because it is practically unfeasible to systematically investigate all of these relationships experimentally, we complemented experiments with muscle modelling. After demonstrating that our model provides an excellent description of the type of contractions examined here, we used it to obtain further insight into these relationships. We hope that by revising the first paragraph of the Introduction and the last section of the Discussion, the main motivation for our study is clear.

Comment: *5. Assuming geometric scaling, one could expect that the time scale of fibre contraction would linearly scale with fibre length, and therefore, given the idea that indeed strain rates dominate the AMPO characteristics you have determined, the present AMPO plots (of rats) versus frequency would expectedly be invariant for animals of different size if plotted versus the product of (characteristic movement) frequency, which expectedly scales inversely with time, and optimal fibre length, instead of just frequency; also, AMPO normalised to body mass would expectedly be invariant. May you comment on expected allometry of AMPO characteristics?*

Response: Indeed, how AMPO depends on properties as fibre length etc. depends on the assumptions when scaling the model. For example, when increasing or decreasing optimal CE length (L_{CE}^{opt}) the question is whether the force-velocity relationship changes as well. In case the shape of the force-velocity relationship remains similar, but only the maximal shortening velocity

changes (thus: normalised to L_{CE}^{opt} it stays the same), then only the muscle length excursion scales linearly with the in- or decrease with optimal fibre length (thus: staying the same in terms of normalised length change). However, when not adjusting/scaling the force-velocity relationship this would affect the optimal values of cycle frequency, FTS and MTC length excursions. We have performed such sensitivity analysis, where these values were relatively robust again realistic changes in muscle properties/muscle parameter values. However, including a detailed presentation of this sensitivity analysis in the current manuscript would further increase its length, which is already substantial.

As an aside, but potentially to your interest: we also explored the effect of muscle fibre type. To do so, we assumed that the activation and deactivation time constants scale proportionally with the maximal shortening velocity, which is a reasonable approximation based on findings by Close (1972). Specifically, if the activation and deactivation time constants are scaled by a factor C, the maximal shortening velocity (vcemax) decreases by a factor 1/C.

Under this assumption, both AMPO and the AMPO-optimal cycle frequency scale linearly with 1/C, whereas the AMPO-optimal FTS and CE/MTC length excursion remain unchanged. This can be explained as follows: 1) starting from the same initial level of Ca^{2+} between the myofilaments (γ), it takes 1/C times as long to reach a given γ (assuming a non-steady state situation and similar CE length as a function of normalised time; where normalised time = 1/cycle duration). 2) the CE force @ vce=x/C in the scaled version is similar to the CE force @ vce=x in the unscaled version.

Consequently, when the cycle frequency is also scaled by 1/C, the activate state as a function of normalised time remains similar between the scaled and unscaled version. At the same time, the force–velocity effects remain the same, if the CE length as a function of normalised time is unchanged. Because the force–length relationship is not affected by muscle fibre type, this leads to similar CE length and thus similar CE force over normalised time.

Therefore, given a solution for one muscle, it is straightforward to derive maximal AMPO and the corresponding optimal CE length over time for any scaling factor C, under the assumptions that (1) activation and deactivation time constants scale linearly with 1/vcemax, and (2) all other muscle model parameters remain constant.

While scaling for muscle fibre type might be straightforward, it is obviously not the only muscle property that differs between (individual) muscle of various species. Such differences may explain, for example, variation in optimal muscle length excursion expressed in %Lceopt: 22% for mouse m. soleus and 16% for mouse m. extensor digitorum longus (Askew & Marsh, 1997), where we found a value of 58% for rat m. gastrocnemius medialis. Thus, although optimal muscle length excursion (and cycle frequency) appears to depend on muscle-specific properties, the optimal FTS appears to be relatively robust to such differences in muscle properties. As explained in the ms (section “The optimal FTS was relatively constant across all SSC conditions”), the optimal FTS reflects mainly a trade-off between (the negative effect on AMPO of) the concentric CE force-velocity relationship and the excitation dynamics. Since both are strongly (and similarly) linked to fibre type, these (negative) effects are similar across different muscle fibre types (see above). Consequently, the optimal FTS for AMPO was found to be rather similar across different muscles (~0.85 for mouse soleus and edl, see Askew & Marsh, 1997; ~0.85 for rat medial gastrocnemius, see this study and ~0.80 for human quadriceps femoris; see Reuvers et al. 2025).

Action: Because the manuscript is already quite long, we believe that this topic of scaling is better suited for a separate study. The strong agreement between our model and experimental data provides a solid basis for such future work, which we are currently pursuing. Nevertheless, we slightly revised second paragraph of the section "The optimal FTS for AMPO was relatively constant across all SSC conditions" to address the generalisability of optimal FTS across different muscle (properties), it now reads:

"The optimal FTS reflects mainly a trade-off between the CE concentric force–velocity relationship and excitation dynamics. The negative effects of both the CE force–velocity relationship and excitation dynamics on net mechanical work per cycle increase with cycle frequency. Yet, the fact that the optimal FTS remains relatively constant across all cycle frequencies studied suggests that these negative effects increase to a similar extent with cycle frequency (Figure 9). Moreover, because the negative effects of the CE force–velocity relationship and excitation dynamics on AMPO are both strongly and comparably linked to muscle fibre type (Close, 1972), their combined influence is likely relatively similar across muscles with different fibre type compositions. Consequently, it is perhaps not surprising that the optimal FTS for AMPO remains relatively consistent across different muscles (~0.85 for rat *m. gastrocnemius medialis* in this study; ~0.85 for mouse m. soleus and *m. extensor digitorum longus* [see 8] and ~0.80 for human *m. quadriceps femoris*, see [see 26]). Together, these findings suggest that the optimal FTS is not only relatively constant across all SSC conditions, but also across different species and muscles (with varying fibre types)."

Word corrections and other minor comments

Comments:

l.130: Remove "data".

l.176: 'used

l.207: I could not unambiguously read from the text whether the excursion (amplitude) is meant peak-to-peak, rather than half that value. Yes, if one has taken a look at LCE,opt values and Fig.2, then it is clear it must be peak-to-peak. Yet, please clarify explicitly here to help the reader: "(peak-to-peak, see Figure 2)".

l.256: Sure you want to say "CE length as sensitivity to Ca2+ increases with CE length"? Shouldn't it be "sensitivity of active state q to Ca2+ increases with CE length"?

l.257: "The concentration 'of' free Ca2+"

l.265: "based on their" instead of "using on their"

Fig.5: In legend, "The first 't'rial with"; I suggest to use the same AMPO range for all sub-panels (0-120mW).

Fig.6: In legend, please, explain that the time of stimulus is depicted by the horizontal bars.

Fig.7: In legend, it should be "Each dot represent's'".

l.402: "imposing any constraints 'on' MTC length"

l.520-521: "due 'to' a higher shortening"

l.533: SEE slack length and compliance are the only properties modelled, thus, it is enough to write "...SEE properties, its slack length and elastic compliance." [or simply "elasticity"]

l.718: 'cost function'

Response/action: Thank you for catching these. We have all changed this according to your recommendations.

Comment: *l.312-313: Your F_{CE}^{max} CE values (sum of GM and GL) are practically identical to what has been given in (16) (30N), as are your animal masses; also, your GMs' F_{CE}^{max} values are consistent with (17) and (18); you may or may not use these sources as validation, just a suggestion.*

Response/action: Thank you for the suggestion. We reviewed the cited studies and agree that there is good agreement between the summed maximal isometric force of GM and GL in our study and the values reported by Christensen et al. (2017). The GM forces reported by Ettema & Huijing (1994) and Zuurbier & Huijing (1993) are slightly lower, which may be explained by their somewhat lower animal masses.

In general, however, comparing maximal isometric force across studies is challenging, not only due to differences in animal mass (and more importantly muscle size), but also due to methodological factors. For example, stimulation frequency influences maximal isometric GM force (e.g. de Haan, 1998): values are relatively constant above ~120 Hz, whereas lower stimulation frequencies result in reduced maximal isometric force. While most studies used a 100 Hz stimulation frequency, those using lower frequencies (e.g. Zandwijk et al., 1996; Meijer et al., 1997) may report lower isometric GM forces, making direct comparisons less straightforward. In addition, other methodological choices, such as whether myofascial force transmission is minimised by surgical procedures, can also influence measured force values. Taken together, these factors should be considered when comparing maximal isometric GM and/or GL forces across studies.

The main purpose of the paragraph referred to by the reviewer was to demonstrate that the effect of GL+PL on GM was negligible. We feel that including a more detailed comparison with previously reported force values would shift the focus away from this central message. Consequently, we have chosen not to include these additional references in the revised manuscript.

	**Mass [g]**	**Stim freq [Hz]**	**Fmax [N]**
Our study	365, 410 & 407g	100	GM: 15.5, 14.1, 17.0 N
Christensen et al. (2017)	413 ± 16	100	GM+GL: 30 N
Ettema & Huijng (1994)	256-333	100	GM: ~14 N (typical value)
Zuurbier & Huijng (1993)	280-308	100	GM: 11.9 ± 0.2 N
Zandwijk et al. (1996)	Not given	20, 40, 60 & 80	GM: 13.3, 13.8 & 12.3 N
Rijkelijkhuizen et al. (2005)	250-289	200	GM: ~10 N
Meijer et al. (1997)	294 ± 5	80	GM: 8.5 ± 0.4
De Haan (1998)	240-280	60, 80, 120, 160, 200, 250, 300 & 400	GM: ~ 11 N
Ettema & Huijng (1992)	233-329	100	GM: 11.0 ± 0.7 N
Bemis et al. (2024)	Not given	180	GM: 17.9 N

Comment: *Table 1: In legend, please, add ”of rat GM” after ”properties”; I think, it would also be useful to give the three specimens' body mass values in this legend, and, if determined or estimated, muscle mass or volume in the table. Finally, for easy comparison to a lot of other articles on rat muscles, it would be nice to also explicitly present the values of Hill's relative parameters arel and brel under ”Derived metrics”.*

Response/action: We have changed the caption of Table 1. Unfortunately, we did not measure/estimate the muscle mass of each specimen. Given that both absolute (a & b) and relative (a^{rel} & b^{rel}) parameters convey the same information (together with F_{CE}^{max} and L_{CE}^{opt}), we opted to report only a^{rel} & b^{rel} in the revised table.

Comment: *l.380-385: According to what I said at the end of REMARKS 1., you should comment on the phase shift visible in Fig.6.*

Response/action: We revised the caption of Figure 6 to explicitly note that the model predicts a slower relaxation of GM force compared to the experimental measurements.

Comment: *l.390-392: See REMARKS 5. (scaling).*

Response: Please see our response to point #5.

Comment: *l.533-535: As SEE is modelled purely elastic, this sentence just formulates the necessary consequence, not depending on specific SEE parameter values, thus, it should simply write: ”Due to SEE elasticity, MTC velocity is always higher (i.e. less negative or even positive) than that of CE during force development (SEE is loaded), and lower (more negative) during force relaxation (SEE is unloaded).”.*

Response/action: We agree that this is indeed not dependent on SEE properties and therefore the phrase “For typical SEE properties” might be a misleading. We have rewritten this sentence to:

“Due to SEE elasticity, MTC velocity should be higher (i.e., less negative or even positive) than that of CE during force development, and lower (i.e., more negative) during force relaxation to maximise AMPO (Figure 10E)”

Comment: *l.557: Shouldn't it be ”before MTC shortening” instead of ”before MTC lengthening”?*

Response/action: Yes, that is correct. Thanks for catching this, we have changed it.

Comment: *l.713-714 (also applies to l.299-301): I find this sentence confusing, as the dynamic load-release interaction between CE and SEE (and also PEE, for longer CE lengths) definitely impacts the overall MTC AMPO (10). You ascertain this fact yourself in l.536-550. Still, I understand what you mean, and accordingly I suggest to change the second part of the sentence into ”... therefore only the time course of CE, rather than MTC, length changes was AMPO-*

optimised to simplify the mathematical problem." Moreover, please, add a sentence why minimising the cost function (Eq.(1)) maximises AMPO.

Response/action: We agree that this sentence is somewhat confusing. Taken together with the comment below we have changed this section to improve clarity.

Comment: *l.720-722: Something is confusing here. I try to break it down. If the sentence in l.720 is correct, then Eq.(2) is really ONE equation (rather than a coupled system) $\dot{x}(t)=\left(\dot{\gamma}(t)\,\dot{L}_{CE}^{rel}(t)\right)^T$, i.e. on its left side it should simply be $\dot{\gamma}(t)$, and you should generally remove the symbol x also on its right side, i.e. write $f\left(L_{CE}^{rel}(t),\gamma(t),u(t)\right)$. Moreover, $STIM(t)$ would be the ONLY (control) input to the system, and $F_{CE}^{rel}(t)$, being a function of both states —AND $\dot{L}_{CE}^{rel}(t)$ immediately known from $L_{CE}^{rel}(t)$ always being given as varied by the optimisation—, would rather be an output, i.e. part of the solution to the optimisation problem. That is, formulating the problem as $F_{CE}^{rel}(t)=F_{CE}^{rel}\left(L_{CE}^{rel}(t),\dot{L}_{CE}^{rel}(t),\gamma(t)\right)$Am I wrong? The alternative would be, that Eq.(2) is really a system of two equations ('$\dot{x}(t)$'), namely contraction and activation dynamics coupled (i.e. the whole Hill-type MTC model), and the inputs would be $STIM(t)$, with $L_{MTC}(t)$ being searched by the optimisation. Is this what you address in l.730 and Eq.(8)?*

Response: We apologise for the confusion, and we would like to thank the reviewer again for studying our paper so carefully. It is rather difficult to explain without going in (too) much detail. It is mostly difficult because model descriptions and computational approaches to solve our Optimal Control problem are mixed (and may be quite counter-intuitive). In addition, the optimisation problem can be implemented in many ways within an optimal control framework. Furthemore, we now see that our explanation left "room for improvement".

In our formulation described in version1 of the ms, we opted for the following formulation: a Hill-type model with states L_{CE}^{rel} and γ and $STIM(t)$ as the first input. Traditionally (and also used in the forward simulation described in Section "Imposed stretch-shortening cycles – constant MTC shortening/lengthening velocity"), MTC length over time is used as the second input. Given that we only explicitly modelled/optimised CE, several (other) options are theoretically possible for the second input, such as CE force and CE velocity. In version1 of the ms, we described that CE force was used as an input. In that case, CE velocity could be computed using the CE force–velocity relationship, based on the active state (determined by γ and CE length), isometric CE force (determined by CE length) and CE force. The resulting CE velocity should then satisfy the dynamic constraint that was described in eq. 2 of version1 of the ms. Thus, given the formulation above, line 720 of version1 of the ms should have been: "The solution had to satisfy the differential equation describing the <u>contraction and</u> activation dynamics:". This is what we initially attempted to describe in our ms.

However, an alternative is possible, which is identical in outcome, but perhaps easier to understand. Since we choose to only explicitly model CE, we could also choose to use L_{CE}^{rel} as an input to the model, rather than as a state variable. In this case, the inputs to the model are $L_{CE}^{rel}(t)$ and $STIM(t)$, while the only state variable is γ. Since, L_{CE}^{rel} over time is known (for every iteration in our optimal

control optimisations), CE velocity can be computed as the time-derivative of CE length. Using CE velocity, CE force could then be computed using the CE force–velocity relationship.

Action: In our revised manuscript we choose to describe our approach as discussed in our response (thus: $L_{CE}^{rel}(t)$ and $STIM(t)$ as inputs and γ as the only state) as in hindsight we consider this formulation more straightforward. In addition, we tried to explain better the rationale behind only optimising CE length over time and calculating MTC length in a second step. The revised text now reads:

"One of the aims of this study was to identify the optimal MTC length over time for maximal AMPO, when MTC length over time is entirely free to take any form. To predict this, we used a Hill-type MTC model. In our Hill-type MTC model, we assumed that both SEE and PEE were purely elastic elements (e.g., Anderson and Pandy, 1999; Soest and Bobbert, 1993). Therefore, during fully periodic SSC neither PEE nor SEE contributed to AMPO of the MTC. This also means that neither PEE nor SEE influences the optimal CE length over time that yields maximal AMPO over a full periodic SSC. We used this to simplify the optimisation problem. We reformulated to model such that we considered a Hill-type model of CE only. As such, CE length was no longer a state variable, but an input to the model. The model therefore had two inputs: CE length and muscle stimuation ($STIM$) over time. The only state variable was γ, the normalised intrafilament Ca^{2+} concentration. Given this formulation, CE length, $STIM$ and γ were known at each time instant during the optimisation process. Consequently, CE velocity can be calculated by taking the time-derivative of CE length. Using CE velocity, CE force could then be computed using the CE force–velocity relationship. MTC length over time was subsequently obtained by taking the following steps: 1) PEE force was calculated from PEE length (which equals CE length); 2) SEE force was calculated by taking the sum of CE and PEE force; 3) SEE length was calculated from SEE force; 4) MTC length was calculated as the sum of CE and SEE length. By computing MTC length over time via these steps, we could also impose MTC length excursion.

The optimisation problem then was to find periodic CE length, STIM and γ over time for maximal AMPO. Optimisations were performed for 1) imposed cycle frequencies (1.0–6.0 Hz in 0.5 Hz steps); 2) imposed FTS values (0.05–0.95 in 0.05 steps) and 3) imposed MTC length excursions (2–11 mm in 1 mm steps). For all optimisation, the following cost function was maximised (note that, in our formulation, CE shortening corresponds to negative CE velocity):

$$J = -\int_{t_0}^{t_0+T_c} F_{CE} \cdot v_{CE}$$

where t_0 denotes the initial time and T_c denotes the cycle time

The solution had to satisfy the differential equation describing the excitation dynamics:

$$\dot{\gamma}(t) = f(\gamma(t), STIM(t))$$"

Response to reviewer #2

Outline

Comment: *In this study, the authors explore the effects of shortening vs lengthening time on maximal power during cyclical contractions with in-vivo and modeling approaches. This question has relevance across species from bird flight to human sport performance. The in-vivo work is*

extremely thorough as well as innovative. Likewise, the modeling work is state of the art and provides an excellent approximation to the in-vivo data. Having worked in both areas myself, I can say that this is one of the most impressive investigations I have ever reviewed. It will definitely be assigned reading in my muscle physiology course.

I have just a few minor comments for the authors' consideration:

Response: We are honoured that the reviewer considers this to be one of the most impressive investigations they have reviewed and that our manuscript may be used in the reviewer's muscle physiology course. We also thank the reviewer for the valuable suggestions, which we have used to improve the manuscript.

Minor comments

Comment: *Line 64: The word optimum is left dangling. Optimum velocity? Trajectory? Please clarify.*

Response/action: Thanks for catching this. We have changed this sentence, it now reads:

"Consequently, it remains unclear how much more AMPO the muscle could produce if the muscle fibre length over time would be optimal for AMPO."

Comment: *Line 136: Consider an alternative work to killed such as euthanized.*

Response/action: We have changed "killed" into "euthanised".

Comment: *Line 218: Two "at"s in a row.*

Response/action: Thanks, we have removed one "at".

Comment: *Line 271: Intraclass correlation might provide a more robust comparison of model and experimental data than plain correlation.*

Response: We agree that intraclass correlation coefficients (ICC) provides a useful measure of agreement when the aim is to assess how closely model predictions match experimental values in absolute terms. In the present study, however, our primary objective was to evaluate how well the model captures the relationship between SSC parameters and the resulting changes in AMPO, rather than the absolute agreement in the magnitude of AMPO. For this reason, we have chosen to report Pearson r^2 values.

Comment: *Lines 503-528: The discussion might be strengthened somewhat if the authors related the interaction of cycle frequency and length excursion to work in human cycling crank length studies which also show strong interactions.*

Response/action: Thanks for this suggestion. We have added an extra sentence regarding the interaction between cycle frequency and crank length in human sprint cycling. To do so, we also had to slightly adjust the text after it:

"A similar interaction between cycle frequency (i.e., cadence) and crank length—which influences MTC length excursion—has also been observed in human sprint cycling (e.g. Martin & Spirduso, 2001), where shortening and lengthening durations of individual MTCs are not necessarily equal. However, to our knowledge, this interaction has not been systematically examined in controlled SSCs with systematically varied unequal shortening and lengthening durations; although Askew and Marsh (1997) briefly noted this interaction, they did not present the relevant data."

Martin J.C., Spirduso W.W. (2001). Determinants of maximal cycling power: crank length, pedaling rate and pedal speed. European Journal of Applied Physiology **84(5)**, 413-8.

Comment: *Line 577: Human should probably be Humans.*

Response/action: Thank you for catching this. This section has been substantially revised and rewritten in the revised manuscript, which has resolved this issue.

Comment: *Lines 13-25 and Figure 1: This paragraph and figure caption provide an excellent description of work loops! Given the emphasis on work loops in the introduction, I was surprised that results were not presented as work loops. I realize the results section is already quite large and thorough, but I wonder if the authors would consider including a few work loop examples for results and discussion.*

Response/action: Thank you for the compliment! Originally, we did not include work loops because we have so many of them (~50 various SSC conditions measured experimentally for each rat) and 1000s of model predictions. Nevertheless, we realise it is somewhat 'strange' not to include any work loop. For this reason, we have revised Figure 6 to include two additional panels showing representative work loops from both experimental measurements and corresponding model predictions.
